# Androgen receptor profiling predicts prostate cancer outcome

**DOI:** 10.15252/emmm.201505424

**Published:** 2015-09-27

**Authors:** Suzan Stelloo, Ekaterina Nevedomskaya, Henk G van der Poel, Jeroen de Jong, Geert JLH van Leenders, Guido Jenster, Lodewyk FA Wessels, Andries M Bergman, Wilbert Zwart

**Affiliations:** 1 Division of Molecular Pathology, The Netherlands Cancer InstituteAmsterdam, The Netherlands; 2 Division of Molecular Carcinogenesis, The Netherlands Cancer InstituteAmsterdam, The Netherlands; 3 Division of Urology, The Netherlands Cancer InstituteAmsterdam, The Netherlands; 4 Division of Pathology, The Netherlands Cancer InstituteAmsterdam, The Netherlands; 5 Department of Pathology, Josephine Nefkens Institute, Erasmus Medical CenterRotterdam, The Netherlands; 6 Department of Urology, Josephine Nefkens Institute, Erasmus Medical CenterRotterdam, The Netherlands; 7 Division of Medical Oncology, The Netherlands Cancer InstituteAmsterdam, The Netherlands

**Keywords:** androgen receptor profiling, ChIP-seq, companion diagnostics for prostate cancer, FAIRE-seq, treatment prediction

## Abstract

Prostate cancer is the second most prevalent malignancy in men. Biomarkers for outcome prediction are urgently needed, so that high-risk patients could be monitored more closely postoperatively. To identify prognostic markers and to determine causal players in prostate cancer progression, we assessed changes in chromatin state during tumor development and progression. Based on this, we assessed genomewide androgen receptor/chromatin binding and identified a distinct androgen receptor/chromatin binding profile between primary prostate cancers and tumors with an acquired resistance to therapy. These differential androgen receptor/chromatin interactions dictated expression of a distinct gene signature with strong prognostic potential. Further refinement of the signature provided us with a concise list of nine genes that hallmark prostate cancer outcome in multiple independent validation series. In this report, we identified a novel gene expression signature for prostate cancer outcome through generation of multilevel genomic data on chromatin accessibility and transcriptional regulation and integration with publically available transcriptomic and clinical datastreams. By combining existing technologies, we propose a novel pipeline for biomarker discovery that is easily implementable in other fields of oncology.

## Introduction

Prostate cancer is the second most common cancer in men, with worldwide more than one million new patients diagnosed and 300,000 deaths annually (Torre *et al*, 2015). When the disease is confined to the prostate, patients can be treated with prostatectomy and/or radiotherapy with a curative intent. However, the disease recurs in 30% of patients, for which there is no cure (Amling *et al*, 2000).

Androgen receptor (AR) plays a pivotal role in prostate cancer development and progression, by mediating transcription of pro-mitotic genes, including UBE2C and cyclin D, resulting in prostate cancer cell proliferation (Xu *et al*, 2006; Wang *et al*, 2009). Upon androgen stimulation, AR dissociates from its chaperones and translocates to the nucleus (Brinkmann *et al*, 1999). Subsequently, AR binds at distinct genomic regions to mediate expression of directly responsive genes, ultimately leading to tumor cell proliferation (Itkonen & Mills, 2012). AR binding requires accessible chromatin, which is facilitated by pioneer factors, including FOXA1 and GATA2 (Bohm *et al*, 2009). Chromatin-bound AR subsequently recruits coactivators and corepressors which facilitate or repress its transcriptional activity, respectively (Shang *et al*, 2002). Differential expression levels of AR pioneer factors and coregulators correlate with clinical outcome (Bohm *et al*, 2009; Sahu *et al*, 2011), implicating deregulation of the androgen-signaling axis in prostate cancer development and progression.

Androgen deprivation therapy (ADT) abrogates androgen signaling either through diminishing androgen synthesis or through competitive binding of the receptor, both resulting in a reduction of transcriptional activity of the AR. Patients with failed salvage therapy or metastatic prostate cancer are treated with ADT as a first-line palliative treatment (Heidenreich *et al*, 2014a). Moreover, adjuvant ADT improves the chances of cure for patients treated with radiotherapy (Pilepich *et al*, 2001; Bolla *et al*, 2009). Still, not all radiotherapy-treated patients will benefit from adjuvant ADT (Roach, 2014). After prostatectomy, there is no conclusive evidence for benefit from adjuvant ADT (Zincke *et al*, 2001; Dorff *et al*, 2011; Miocinovic *et al*, 2011; Siddiqui *et al*, 2011; Briganti *et al*, 2012; Schubert *et al*, 2012; Tsurumaki Sato *et al*, 2014), but it is not unlikely that a subgroup of patients may benefit from adjuvant ADT after prostatectomy. Early identification of high-risk patients would be of substantial clinical relevance, so that these patients could be monitored more closely.

D’Amico *et al* (1998) developed a classification system based on clinical parameters (PSA, Gleason and clinical staging) to group men in low, intermediate, and high risk of relapse after therapy with curative intent. Limitation of this classification is the lack of integration with multiple risk factors and genomic data, which could provide more personalized risk assessment. Besides clinical risk stratifications, a number of different genomic classifications have been developed that enable the identification of high-risk patients (Irshad *et al*, 2013; Lalonde *et al*, 2014; Ramos-Montoya *et al*, 2014). However, no such genomic risk assessment biomarkers are currently adopted in routine clinical practice.

ADT can keep metastatic disease under control for several years, but practically all tumors eventually develop resistance to treatment. The majority of ADT-resistant tumors maintain active AR signaling, rendering this pathway a legitimate target for a second-line endocrine therapy (Valenca *et al*, 2015). Numerous novel anti-androgens and androgen depleting agents are being introduced into the clinic, including enzalutamide (MDV3100), ARN-509, and the CYP17 inhibitor abiraterone (Potter *et al*, 1995; Tran *et al*, 2009; Clegg *et al*, 2012). Unlike older anti-androgens (bicalutamide and flutamide) (Culig *et al*, 1999; Scher & Sawyers, 2005), the new generation of anti-androgens (e.g. enzalutamide) prevent nuclear translocation of AR and do not exhibit agonistic properties. But despite clinical implementation of these improved inhibitors of AR signaling, response is partial and temporal and tumors inevitably progress into a more aggressive and typically lethal form of prostate cancer (Antonarakis *et al*, 2014). Various mechanisms underlying resistance to abiraterone and anti-androgens are known, including AR overexpression, AR splice variants that confer ligand independent AR transactivation, and alterations in expression and recruitment of AR coregulators (Lamb *et al*, 2014).

AR chromatin binding and expression of AR-responsive genes were found to deviate between androgen-sensitive and androgen-resistant cell lines (Wang *et al*, 2009). Although AR binding profiles in cell lines have been studied extensively, the genomic behavior of AR in human prostate specimens remains largely understudied. AR chromatin binding profiles found in treatment-resistant prostate tumors were also observed in prostate cancer cell lines, and highlighted genes correlated with survival (Sharma *et al*, 2013). Recently, AR binding sites were identified that differentiated normal prostate tissue from cancer, which associated with the onset and progression of prostate cancer (Chen *et al*, 2015). Still, no thorough assessment of AR binding between primary versus resistant tumor specimens has been performed to date.

By comparing chromatin accessibility (Formaldehyde-Assisted Isolation of Regulatory Elements (FAIRE)-seq) and AR chromatin binding profiles (Chromatin Immunoprecipitation (ChIP)-seq) in primary versus ADT-resistant tumors, we identified a distinct gene set that enables stratification of patients with prostate cancer on outcome. With this, our study illustrates that progressive disease yields prognostic information in primary lesions and provides a prognostic gene signature to identify patients with prostate cancer at risk of metastatic relapse after local–regional treatment.

## Results

### Genomics-based pipeline for biomarker discovery and validation

**Figure 1:**
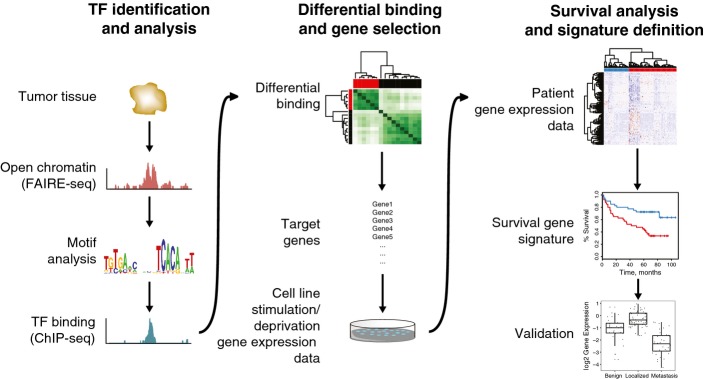
Genomics-based pipeline for biomarker discovery and validation Tissue samples were processed for FAIRE-seq, and transcription factor (TF) motifs in open chromatin regions were analyzed. Selected transcription factor was mapped with ChIP-seq (in this case androgen receptor) to identify sites that are differentially bound between two sample groups. The target genes of the differential binding regions were coupled to gene expression and survival data and further refined into a minimal gene signature, which was validated in a number of gene expression datasets.

By combining existing technologies, we here propose a genomics pipeline for biomarker discovery (Fig1) and showed its application in prostate cancer, aimed at identification of prostate cancer patients with a high-risk of metastatic relapse. Firstly, transcription factor involvement was identified through motif analysis on open chromatin regions. Accessible regions were analyzed to reveal enrichment of a binding motif for a certain transcription factor involved in disease (prostate cancer). Actual transcription factor binding was mapped with ChIP-seq to identify sites that are differentially bound between two sample groups. As a proof-of-principle, we assessed AR chromatin binding profiles in this study. The target genes of the differential binding regions were subsequently coupled to gene expression data in cell lines to uncover genuine involvement of the transcription factor in expression of a distinct gene set. This gene set was subsequently tested for association with survival data of patients, and further refined into a minimal gene signature.

### Genomewide profiling of accessible chromatin regions in prostate tissues by FAIRE-seq

**Figure 2:**
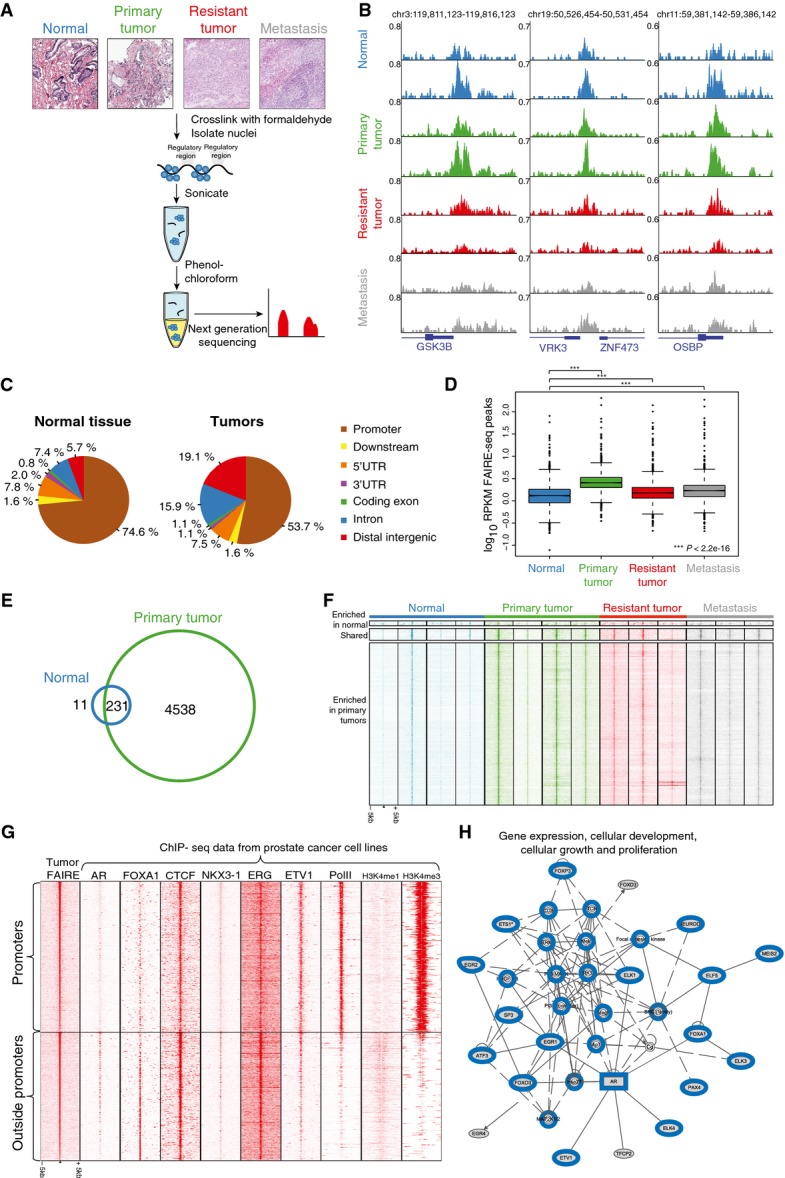
Genomewide profiling of chromatin accessibility in prostate cancer specimens Overview of the experimental design. Representative examples of H&E-stained slides are shown. DNA and proteins were cross-linked using formaldehyde, followed by shearing of the chromatin and phenol–chloroform extraction. The organic phase contains compacted DNA (protein–DNA complexes), while DNA recovered from the aqueous phase represents accessible regulatory regions. DNA from the aqueous phase is further purified and sequenced.

We assessed chromatin accessibility in multiple prostate tissue specimens as well as the changes thereof in prostate cancer development and progression. Four normal prostate tissue samples, four primary tumors, and three ADT-resistant prostate tumors were assessed, as well as three prostate cancer metastases (Fig2A). FAIRE-seq was applied to identify accessible chromatin regions with gene-regulatory functions on a genomewide scale (Giresi & Lieb, 2009). FAIRE is based on phenol–chloroform mediated sample separation, in which accessible chromatin fragments can be separated from the condensed state, effectively enriching for regulatory genomic regions (schematically visualized in Fig2A). Metastases and prostate adenocarcinomas contained more than 70% tumor cells with a Gleason score ranging from 7 (3 + 4) to 10 (5 + 5), while all normal prostate tissues were derived from a healthy region from prostatectomy specimens. Tumor and normal tissues were validated by our pathologists. Clinicopathological parameters are shown in Appendix Table S1. The number of FAIRE peaks identified was highly variable between the tissues, ranging from 50 peaks up to over 13,000 peaks (Appendix Table S2). Figure2B shows four randomly selected representative coverage profiles of accessible chromatin at promoter regions. Over 50% of accessible chromatin sites in healthy and tumor specimens were found at promoter regions (Fig2C), and average signal for each specimen showed clear enrichment of reads at transcription start sites (Appendix Fig S1). Tumor samples showed more enriched chromatin accessibility at both intron and distal intergenic regions, as opposed to normal prostate tissue where FAIRE signal was mainly found at promoters (Fig2C).

Snapshots of accessible chromatin regions as assessed through FAIRE-seq in normal prostate tissue (blue), primary tumor (green), treatment-resistant tumor (red), and metastatic (gray) tissue. Reads are normalized to millions of sequenced reads per sample. Genomic coordinates are indicated.

Genomic distribution of FAIRE peaks in normal and tumor samples.

Boxplots depicting normalized FAIRE-seq read counts (RPKM) in different tissues across the peaks found in at least three samples. Read counts in benign tissue (blue) are lower than in primary tumor (green), therapy-resistant tumor (red) or metastasis (gray) (*P* < 2.2e−16, paired *t*-test).

Venn diagram, visualizing shared and unique FAIRE peaks in normal prostate samples (blue) and primary prostate tumor samples (green).

Heatmap showing raw read count intensity in FAIRE-seq peaks enriched in either normal or tumor samples. The window represents 5 kb around the FAIRE-seq peak.

Heatmap showing raw read count intensity of ChIP-seq signal from multiple cell line datasets (Appendix Table S12 for references and GEO accession numbers) at accessible regions identified in primary tumors (peaks present in at least two specimens) ranked on peak intensity. Top panel depicts promoter regions, and the bottom panel, all other regions. The window represents 5 kb around the FAIRE-seq peak.

Ingenuity pathway analyses, illustrating one of the networks based on motifs found in FAIRE-seq peaks that were present in at least two out of four primary tumors. Genes previously described to be involved in prostate cancer are highlighted in blue (other networks in Appendix Fig S3).

The number of sequenced reads as well as called peaks was lower in normal tissue as compared to any of the tumor samples: three out of four normal tissue samples (75%) had < 25 million reads sequenced, while nine out of ten tumor samples had above 25 million reads sequenced (90%; *P* = 0.04, Fisher’s exact test), and three out of four normal tissue samples had < 1,000 peaks versus two out of ten tumor samples (*P* = 0.09, Fisher’s exact test) (Appendix Table S2). This suggested presence of more condensed chromatin in normal prostate tissue compared to tumor. To confirm this, we compared normalized read count (reads per kilobase per million (RPKM)) in peaks found in at least three FAIRE-seq samples (*N* = 3,010). Read counts were significantly lower in normal tissue as compared to any of the tumor stages (*P* < 2.2e−16, paired *t*-test) (Fig2D). Normalized read count in advanced disease was lower than in primary prostate cancer (*P* < 2.2e−16 for both resistant and metastatic tissue), but still higher than in normal tissue (Fig2D).

To further focus on the differences in tumorigenesis, we considered peaks found in at least two out of four normal tissue samples and two out of four primary tumors. A much larger number of accessible chromatin regions are found in primary prostate cancer compared to normal tissue (Fig2E). This corresponds to a higher raw signal in primary and other tumor stages compared to normal tissue (Fig2F).

To identify transcription factors potentially involved in prostate cancer development, we performed motif enrichment analysis on chromatin regions selectively accessible in tumors. In addition, we analyzed the overlap of accessible FAIRE sites with ChIP-seq data from a multitude of transcription factors in various cells using the ReMap annotation tool (Griffon *et al*, 2015). As expected, multiple motifs of transcription factors involved in prostate cancer pathobiology were found, including ERG (Gasi Tandefelt *et al*, 2014), AR (Lonergan & Tindall, 2011), and its pioneer factor FOXA1 (Robinson & Carroll, 2012) (Table EV1). The motifs are generally conserved between the individuals (Appendix Table S3). Using the ReMap tool, we found overlap of FAIRE sites with ChIP-seq signal from a number of factors from which motifs were found in FAIRE-seq data (e.g. AR, ERG, SP1, ETS1, and others) (Appendix Fig S2). Moreover, at these FAIRE regions a weak but statistically significant correlation was found between motif enrichment and ChIP-seq overlap from the ReMap tool (Spearman ρ = 0.30, *P* = 0.02). CTCF was found to be an outlier with high score of motif and low degree of overlap, while AR, MYC, and ERG showed moderate-to-high overlap and motif score.

**Figure EV1:**
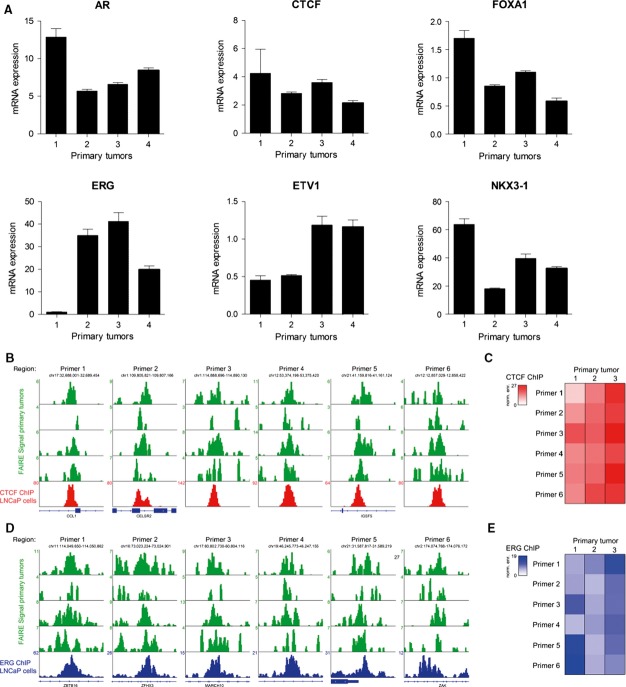
Expression and occupancy of a set of transcription factors corresponding to the identified motifs at FAIRE peaks Expression levels of AR, CTCF, FOXA1, ERG, ETV1, and NKX3-1 relative to TBP in four independent primary tumors. Error bars indicate SD from triplicate analysis.

Subsequently, mRNA expression and DNA binding for multiple of these transcription factors were assessed in prostate tumors and cell lines. For AR, CTCF, ERG, FOXA1, ETV1, and NKX3-1, mRNA expression was confirmed in four primary tumors (FigEV1A). Using publically available ChIP-seq data from LNCaP and VCaP cells, we could illustrate these factors occupying the FAIRE-seq regions in the tumors (Fig2G). As expected, AR binding was enriched at the enhancer-associated accessible regions, while CTCF occupied both enhancers and promoters (Taslim *et al*, 2012). For ERG and CTCF, we further validated binding at a subset of these regions in primary tumor specimens by ChIP–qPCR (Fig EV1B–E). Functional involvement of AR was further evidenced by ingenuity pathway analysis using the list of genes corresponding to the motifs identified in FAIRE-seq peaks of the primary tumors (Table EV1). This analysis yielded functional networks known to be involved in prostate cancer, with one network centered around AR (Fig2H, Appendix Fig S3).

FAIRE-seq snapshots of accessible chromatin regions containing a CTCF motif in four primary tumors (green) and CTCF binding as assessed through ChIP-seq in LNCaP cells (red track, GSE33213).

Heatmap, illustrating ChIP–qPCR-based enrichment of CTCF binding at accessible chromatin sites depicted in (B).

FAIRE-seq snapshots of accessible chromatin regions containing an ERG motif in four primary tumors (green) and ERG binding as assessed through ChIP-seq in LNCaP cells (blue track, GSM1193658).

Heatmap, illustrating ChIP–qPCR-based enrichment of ERG binding at accessible chromatin sites depicted in (D).

### Distinct genomewide AR binding pattern in primary and resistant tumors

**Figure 3:**
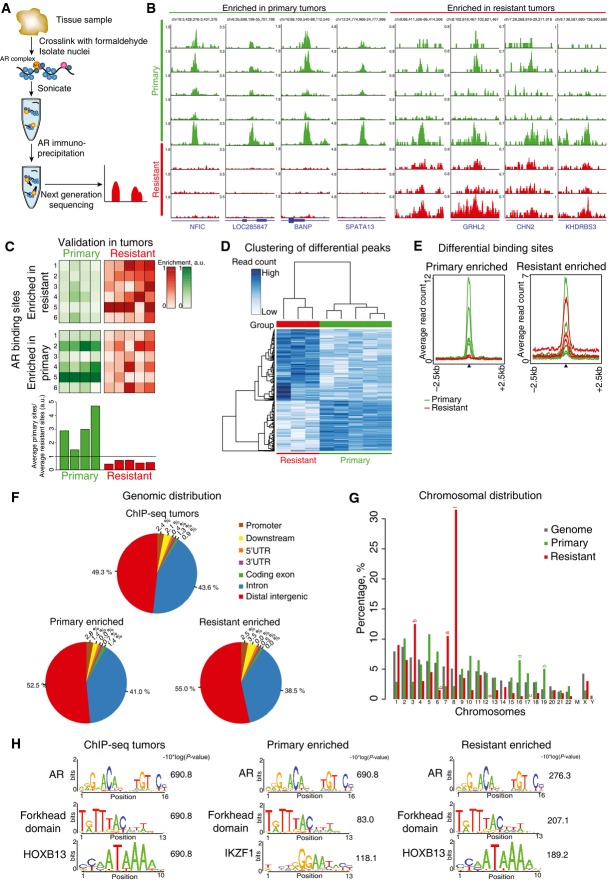
Distinct AR binding profiles in primary and treatment-resistant tumors Overview of experimental design. DNA and proteins were cross-linked using formaldehyde, followed by shearing of the chromatin. Immunoprecipitation for AR was performed after which isolated DNA fragments were sequenced.

Since AR and its interactors were enriched at FAIRE-seq regions (Fig2G), we next performed AR ChIP-seq on five primary and three treatment-resistant tumor specimens (Fig3A). The number of reads and AR binding events is shown in Appendix Table S4. The total number of identified AR binding events greatly varied between the tumor samples (Appendix Table S4), which is consistent with previous nuclear receptor ChIP-seq in prostate and breast tumor samples (Ross-Innes *et al*, 2012; Jansen *et al*, 2013; Sharma *et al*, 2013). Specifically, our AR binding sites varied from 238 up to 17,511 per tumor sample, which is in the same order of magnitude as in Sharma *et al*, with 300–8,500 per tumor sample (Sharma *et al*, 2013).

Snapshots of AR binding events differentially enriched in either primary (green) or resistant (red) tumors. Reads are normalized to millions of sequenced reads per sample. Genomic coordinates are shown.

Heatmap illustrating AR ChIP-qPCR validation in independent tumors. Intensity of the color corresponds to ChIP–qPCR enrichment (see scale bar). Binding sites identified as enriched in either primary or treatment-resistant tumors were tested. Average enrichment in primary tumors was divided over average resistant enrichment values to determine the ratio, as is visualized in a barplot (bottom panel).

Differential binding analysis of AR chromatin binding regions, selectively enriched in primary tumors (green) or treatment-resistant (red) tumors.

Average read counts for AR binding events, selectively enriched between primary (green) or resistant (red) tumors. Data are centered at AR peaks, depicting a 2.5-kb window around the peak.

Genomic distributions of AR binding sites shared between tumors or enriched in primary and resistant tumor tissue.

Distribution of AR binding sites enriched in primary (green) and resistant (red) tumors, by chromosome (%). *P*-values (one-sided binomial test) for significant enrichment relative to the entire genome (gray): (a) *P* = 0.001, (b) *P* = 0.002, (c) *P* = 0.003, (d) *P* = 0.005, (e) *P* = 9e−13, and (f) *P* = 2.5e−13.

Motif enrichment analysis for AR binding sites shared between tumors or enriched in primary and resistant tumor tissue. Top motifs are shown.

Unlike in FAIRE data, the majority (over 90%) of AR ChIP-seq peaks are present either in intronic or distal intergenic regions throughout the genome (Figs2C and 3G), consistent with profiles identified in cell lines (Yu *et al*, 2010; Asangani *et al*, 2014) and tumors (Sharma *et al*, 2013). Intra-tumor heterogeneity effect was limited, as assessed on two independent sections from the same tumor specimen, with 87% overlap of AR binding sites between the biological replicates and high correlation of peak read counts (*r* = 0.76) (Appendix Fig S4). Overlap of AR ChIP-seq replicates was comparable to previously described cell line data (Bolton *et al*, 2007; Jia *et al*, 2008). AR binding sites selectively enriched in resistant or primary tumors could be observed (Fig3B), as validated by AR ChIP–qPCR analyses on additional four primary and five resistant tumors (Fig3C). Ratios of average enrichment for “resistant enriched” over “primary tumor enriched” regions were determined, analogous to what we performed before for breast cancer samples (Jansen *et al*, 2013), showing consistent subclassification of patients in the validation set.

To assess differential AR chromatin binding on a global scale between treatment-resistant and primary tumors, differential binding analysis was performed. Peaks present in at least three tumors (*N* = 3,138) were considered. In total, 339 genomic regions show differential AR binding between primary and resistant tumors (for genomic regions, see Table EV2). Tumors clustered according to their group identity (primary or resistant) based on these regions (Fig3D). Differential AR chromatin binding at these sites is further evidenced by the difference in average read counts between the primary and resistant tumors (Fig3E). To assess the reliability of differential AR binding events, the samples were shuffled randomly based on 56 available permutations. Few or no differential peaks were found when the samples were mislabelled, demonstrating the robustness of classification and no overfitting (Appendix Fig S5). To assess whether the identified differential peaks were also relevant in other clinical datasets, we clustered our data with AR ChIP-seq data of primary tumors from others (Chen *et al*, 2015). Three out of four primary tumors from the other dataset clustered together with the primary tumors from our study using the 339 identified AR sites (FigEV2), suggesting a wider applicability of the differential AR binding signature.

**Figure EV2:**
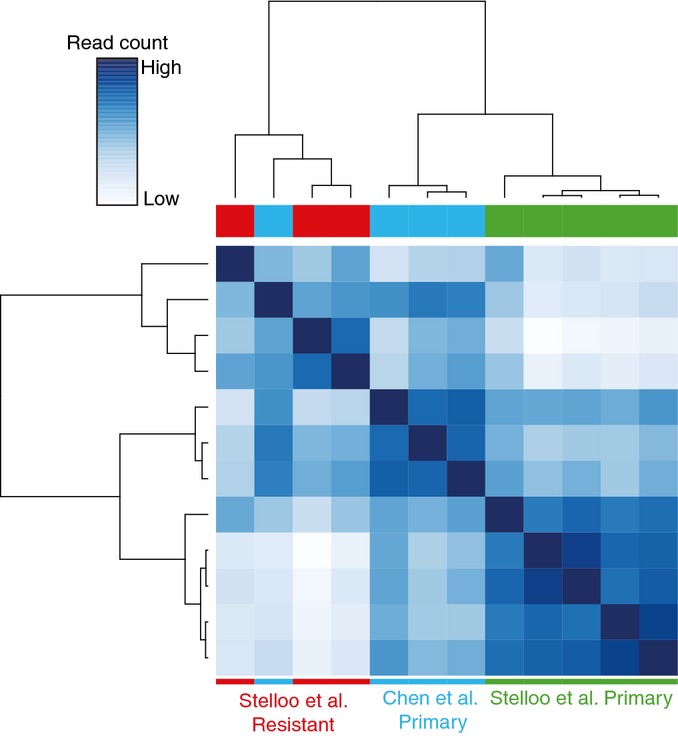
Hierarchical clustering of Stelloo *et al*’s (this manuscript) and Chen *et al*’s data Heatmap of the correlation between the ChIP-seq data from Stelloo *et al* and Chen *et al* based on the 339 differential AR binding sites. Three out of four primary tumors from Chen *et al* cluster together with the primary tumors from this study.

Differential enriched AR peaks are located mostly in intronic or distal intergenic regions of the genome (Fig3F), and primary tumor-associated AR sites are enriched on chromosomes 16 and 19 (Fig3G). In contrast, resistance-associated AR binding sites are enriched on chromosomes 3, 7, and 8, while AR binding at chromosomes 6, 12, and 17 is diminished. The peaks remain enriched in treatment-resistant samples even when the background is subtracted (Appendix Fig S6).

Motif analysis was performed on all AR binding sites (present in at least two tumors), resistance-associated AR binding sites and primary tumor-associated AR sites. As expected, AR binding sites were enriched for AR motifs, as well as for its pioneer factor FOXA1 (Fig3H, Appendix Fig S7, Table EV3). Motif enrichment of the AR coregulator HOXB13 was found selectively in peaks enriched in resistant tumors (Norris *et al*, 2009).

### Genes proximal to altered AR binding sites are AR-responsive in sensitive and resistant cell lines

We identified distinct AR binding regions between treatment-resistant and primary prostate tumors. Possible direct target genes were considered as those with AR binding sites within their body or 20 kb upstream from the transcription start site (Wang *et al*, 2009), yielding 158 genes (Table EV4). The limited number of target genes (158) relative to the 339 AR binding sites can be explained by the functional involvement of multiple AR binding sites to regulate the expression of a single gene, as illustrated at the KLK3 locus (Appendix Fig S8).

**Figure 4:**
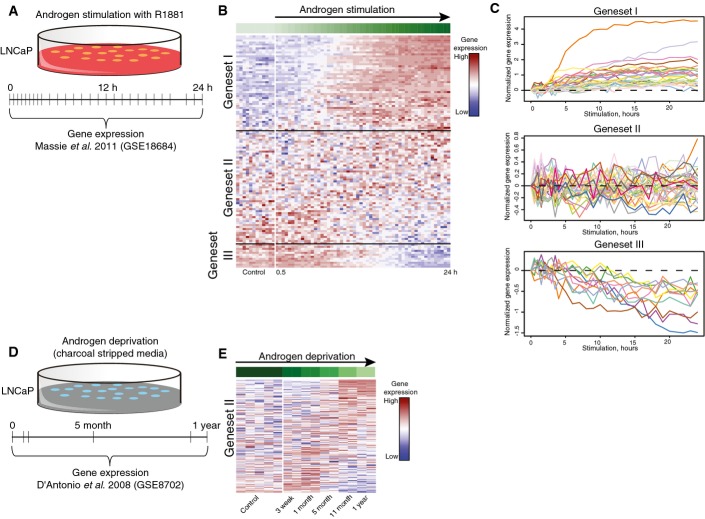
Genes with differential AR binding site are associated with androgen response or androgen resistance Visual representation of the public dataset used (GSE18684; Massie *et al*, 2011). Hormone-deprived LNCaP prostate cancer cells were treated for up to 24 h with 1 nM R1881 and processed for gene expression analyses.

Since genes were identified by alterations of AR binding between primary and resistant tumors, these genes could be AR-responsive in either an androgen-sensitive or androgen-resistant context. To dissect these two possibilities, expression of the 158 putative target genes was explored using a publically available dataset from LNCaP prostate cancer cells treated for up to 24 h with androgen R1881, with samples taken every few hours (Fig4A) (Massie *et al*, 2011). A total of 102 genes out of 158 putative AR target genes were found in the androgen stimulation dataset, represented by 117 expression probes. In order to group genes according to their temporal profiles, we clustered the gene expression data using the Short Time-series Expression Miner (STEM) program. This software utilizes an algorithm that selects potential temporal profiles, assigns genes to those profiles and computes enrichment significance for each profile (Ernst *et al*, 2005; Ernst & Bar-Joseph, 2006). Three significant gene sets were found: I. upregulated in response to R1881 (41 genes); II. not affected by R1881 (49 genes); and III. downregulated in response to R1881 (12 genes) (Fig4B and C, Table EV5). The 49 genes of gene set II were of particular interest, since genes not affected by R1881 treatment could still be associated with acquired ADT resistance. Therefore, expression of these genes was next assessed in a public gene expression dataset from LNCaP cells cultured in charcoal-treated hormone-deprived medium for up to 1 year, mimicking acquired ADT resistance (Fig4D and E) (D’Antonio *et al*, 2008). Out of 49 genes, 48 were annotated in this dataset, corresponding to 164 probes. While not affected by R1881 treatment (Fig4B and C), these genes exhibit differential expression during acquisition of ADT resistance (Fig4E). Expression patterns of gene sets I and III under androgen deprivation conditions are shown in Appendix Fig S9.

Expression of 102 genes with a proximal AR binding site, differentially enriched between primary and resistant tumors, was analyzed. Three subclusters were identified (I, II, III), based on Short Time-series Expression Miner (STEM) clustering analysis. Color scale indicates gene expression level.

Line plots showing expression profiles of the 102 genes in three STEM-based regulatory modules obtained from the androgen stimulated LNCaP expression data as in B.

Visual representation of the public dataset used (GSE8702; D’Antonio *et al*, 2008). LNCaP prostate cancer cells were grown in the absence of androgens for up to 1 year and processed for gene expression analysis.

Heatmap illustrating gene expression of 48 androgen-nonresponsive genes from gene set II in hormone-deprived LNCaP cells (GSE8702).

Cumulatively, by integrating our AR ChIP-seq data with publically available datasets of hormonal response and acquired resistance to ADT, we identified two distinct gene sets associated with “AR response to hormonal stimuli” and “AR response in acquired resistance.”

### Functional and clinical implications of differential AR-binding-affected genes

The AR binding landscape in tumors revealed two clearly defined gene sets, one subset of 53 androgen-responsive genes and the second subset of 49 acquired ADT resistance genes.

**Figure 5:**
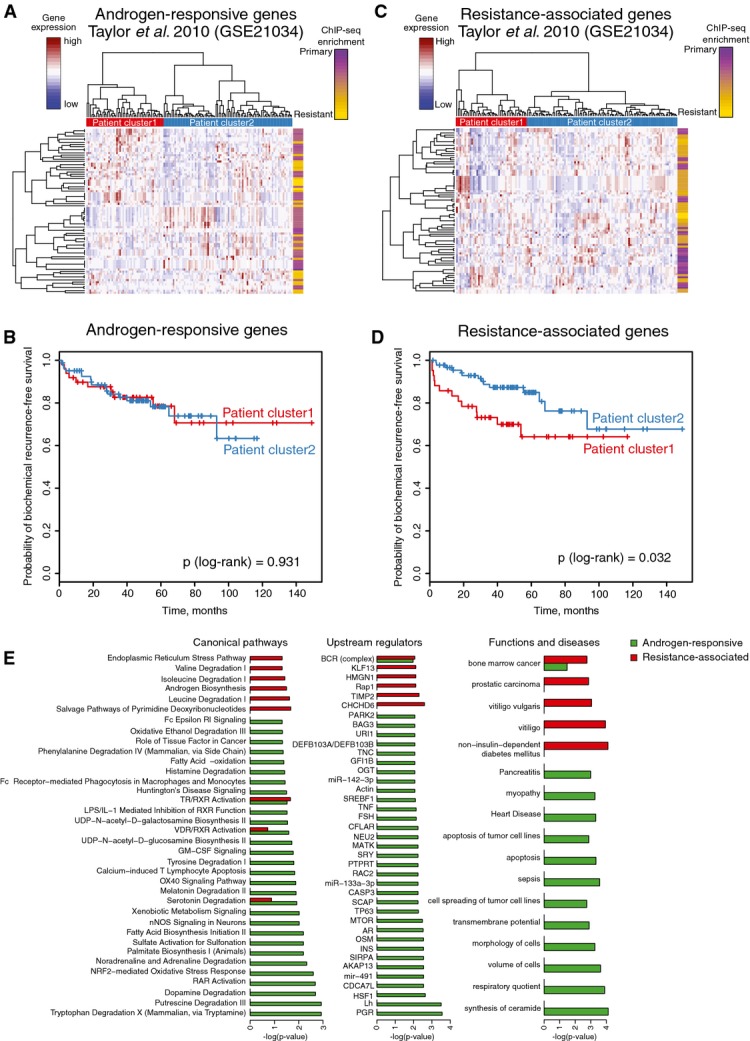
Unsupervised hierarchical clustering and survival analysis based on expression of the selected groups of genes in 131 patients with primary prostate cancer Heatmap of unsupervised hierarchical clustering of 131 patients based on the expression of androgen-responsive gene set.

To couple “androgen-responsive” and “acquired resistance” genes with outcome, gene expression data from 131 primary prostate cancers were analyzed (Taylor *et al*, 2010). Unsupervised hierarchical clustering was performed on the 53 “androgen-responsive” and 49 “acquired resistance” genes separately. The “androgen-responsive” gene set failed to stratify patients on time to biochemical (PSA) relapse (*P* = 0.931, HR = 1.03; 95% CI: 0.47–2.26) (Fig5A and B), while clustering on “acquired resistance” genes did stratify patients (*P* = 0.032, HR = 0.45; 95% CI: 0.21–0.95) (Fig5C and D).

Kaplan–Meier curves of biochemical recurrence-free survival of the two groups of patients identified based on expression of androgen-responsive genes.

Heatmap of unsupervised hierarchical clustering of patients based on the expression of resistance-associated gene set.

Kaplan–Meier curves of biochemical recurrence-free survival of the two groups of patients identified based on expression of resistance-associated genes.

Ingenuity pathway analysis of the two groups of genes (androgen-responsive and resistance-associated). Barplots show significantly enriched pathways, functions, and potential upstream regulators.

Ingenuity pathway analysis illustrated the “androgen-responsive” genes to be involved mainly in cellular metabolic processes and correspond to genes normally regulated by steroid hormone receptors AR and PGR (Fig5E). The “acquired resistance” genes were additionally involved in androgen biosynthesis and contain genes related to prostatic carcinoma (Fig5E).

### Refinement and validation of gene expression classifier

**Figure 6:**
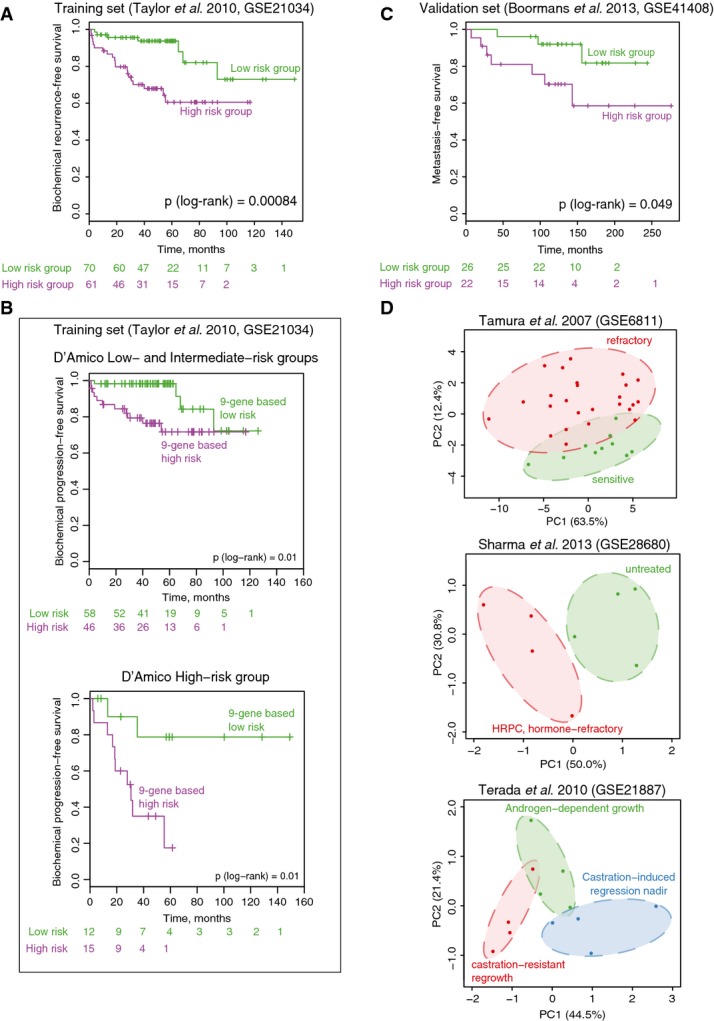
Refinement and validation of gene expression classifier for the survival of patients with prostate cancer Kaplan–Meier survival curves of the patients stratified into two risk groups based on the prognostic index assigned in cross-validated training procedure in the training cohort (Taylor *et al*, 2010).

We identified a set of 49 “acquired resistance” genes that stratify patients with prostate cancer on outcome. Since this list is likely to contain false-positive genes that may jeopardize classification, we identified a core gene set with a prognostic impact through elastic net regularization with double-loop cross-validation. Patients were assigned to two risk groups based on their cross-validated prognostic index (FigEV3), successfully stratifying patients on time to relapse (*P* = 0.00084, log-rank test) (Fig6A). The refined gene set classifier is composed of nine genes: DNER, EXT2, AMOTL1, RBM33, ZBTB20, XBP1, PMFBP1, HSD17B14, and KLF9. Apart from AR (which was differentially enriched proximal to these genes; Fig3), other transcription factors may regulate expression of these nine prognostic genes as well. Therefore, we investigated the overlap of the FAIRE-seq peaks (Fig2) in close proximity of the nine genes (< 20 kb from the transcription start site) with a large collection of public ChIP-seq data from the ReMap tool (Griffon *et al*, 2015). A strong overlap of the identified eight FAIRE-seq peaks was found with known players in prostate cancer including AR, FOXA1, ERG, bromodomains BRD2 and BRD3 and, interestingly, MYC (Appendix Table S5). The latter also occupies a central place in our functional motif analysis (Appendix Fig S3).

**Figure EV3:**
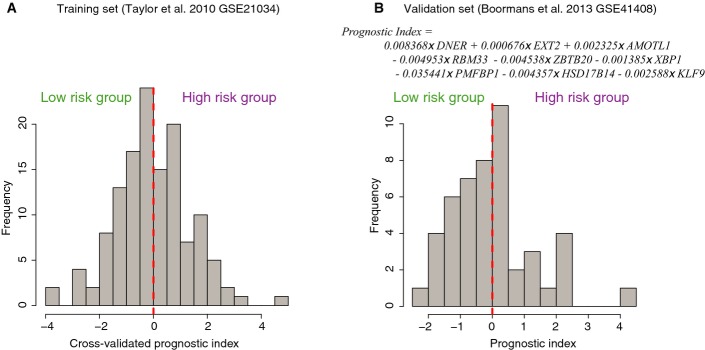
Distribution of the prognostic indices in the training and validation sets Histogram of the prognostic index distribution in the training dataset; patients were assigned to two risk groups based on their positive or negative prognostic index.

Histogram of the prognostic index distribution in the validation dataset.

Kaplan–Meier survival curves for patients stratified based on the 9-gene classifier within low/intermediate- and high-risk groups based on clinical parameters (D’Amico classification).

Validation of the prognostic signature in an independent patient set (Boormans *et al*, 2013). Kaplan–Meier survival curves for patients stratified based on the 9-gene prognostic index.

Principal component analysis score plot based on the expression of the 9-gene signature in three independent expression datasets with hormone-sensitive and hormone-resistant tumors and xenografts.

A prognostic index was assigned to each patient based on the nine genes, calculated as the sum of expression of the nine genes multiplied by their corresponding Cox regression coefficients (Appendix Table S6). This prognostic index is independent from other known prognostic parameters, such as Gleason score, pathologic T stage, lymph node status, and initial PSA (see Appendix Table S7 for patient characteristics), and is significantly associated with time to biochemical recurrence when adjusted for those known prognostic factors (Appendix Table S8). Furthermore, we tested the ability of the classifier to identify patients that develop biochemical recurrence within 5 years after prostatectomy using receiver operating characteristic (ROC) curves. The area under the curve (AUC) value for the 9-gene classifier was slightly higher as compared to an AUC value based on clinical parameters only (0.86 versus 0.83), while combining the genomic classifier with clinical parameters resulted in an AUC value of 0.9 (Appendix Table S9).

One of the most widely used prostate cancer risk assessment classification systems was proposed by D’Amico and colleagues (D’Amico *et al*, 1998). It utilizes clinical TNM stage, preoperative PSA level, and biopsy Gleason score to stratify patients in three risk groups (low-, intermediate-, and high-risk groups). To assess whether our 9-gene classifier is independent from the D’Amico classification, we directly compared the output of D’Amico classification and our 9-gene signature. Low- and high-risk patients as defined by the genomic signature were found in all three D’Amico risk groups (Appendix Table S10), and the prognostic potential of our risk group stratification was independent of D’Amico classification (Appendix Table S11). Since there was no difference in survival between D’Amico low- and intermediate-risk groups in patients from Taylor *et al*’s cohort (FigEV4), we combined low- and intermediate-risk groups together. The 9-gene expression signature was able to further stratify patients in both low/intermediate- and high-risk groups (Fig6B), which further highlights the utility of our 9-gene signature on top of existing classification tools.

**Figure EV4:**
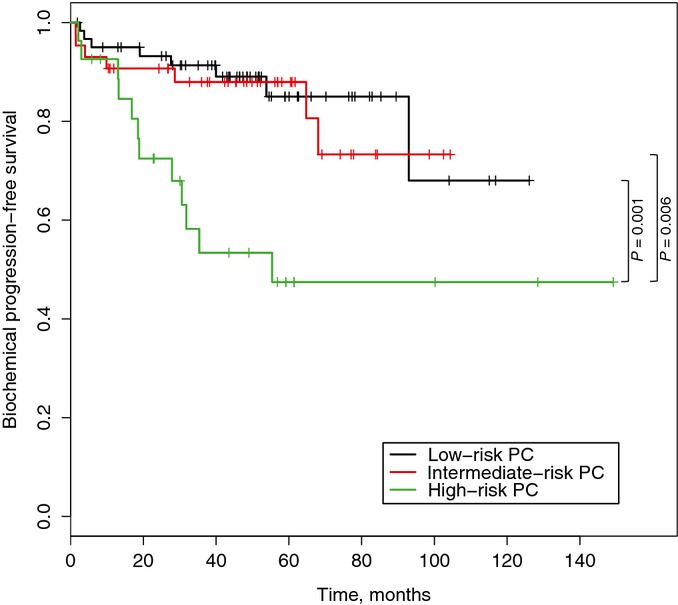
Kaplan–Meier survival curves based on D’Amico classification Kaplan–Meier survival curves of patients from Taylor *et al* cohort stratified into three risk groups based on D’Amico classification using clinical parameters.

For validation of the prognostic signature, we used an independent gene expression dataset from a cohort of 48 patients with prostate cancer (Boormans *et al*, 2013). We applied the coefficients from the trained Cox regression to calculate the prognostic index in the validation set and successfully stratified patients into two risk groups with significant difference in time to metastasis (*P* (log-rank) = 0.049) (Fig6C). Furthermore, we examined two other independent gene expression clinical studies as well as a set of prostate cancer xenografts. We used principal component analysis (PCA) to explore whether expression of the nine genes can separate clinical groups in these datasets, providing new dimensions (principal components) that summarize expression of the nine selected genes. Hormone-sensitive and hormone-refractory tumors and xenografts are separated along either the first or second principal components (Fig6D). With this, we provide evidence that our classifier does not only stratify patients on biochemical relapse (Fig6A and B) and metastasis (Fig6C), but also successfully makes a distinction between hormone-refractory and hormone-sensitive tumors (Fig6D).

Since expression of selected genes is associated with outcome, similar expression differences may be found in metastatic samples. Therefore, we examined expression levels of the nine individual genes in six additional cohorts (Appendix Fig S10, Appendix Table S12) that include benign (normal), primary, and/or metastatic prostate cancer samples. Directionality of gene expression in poor-outcome patients in our discovery set was identical as found in metastatic samples as compared to primary lesions for all six independent cohorts, indicating robustness and consistency (Appendix Fig S10). These data indicate that potential drivers of poor outcome, as identified in primary prostate cancers, are possibly preserved in the metastatic setting.

## Discussion

According to current clinical guidelines, endocrine therapy for prostate cancer is prescribed as an adjuvant treatment after radiotherapy and in the metastatic setting with a palliative intent (Heidenreich *et al*, 2014b). The rationale for not applying endocrine therapies in the adjuvant management of prostate cancer after prostatectomy is provided by multiple clinical trials, which illustrated limited to no clinical benefit of blocking AR function on disease-free survival of the entire population (Zincke *et al*, 2001; Dorff *et al*, 2011; Miocinovic *et al*, 2011; Siddiqui *et al*, 2011; Briganti *et al*, 2012; Schubert *et al*, 2012; Tsurumaki Sato *et al*, 2014). Yet, since ∼30% of patients with prostate cancer do develop a relapse later in life, it is believed that a distinct subgroup of patients may derive benefit from ADT or other subsequent treatment in the adjuvant setting. Above all, patients with high risk of relapse are in need of closer monitoring postoperatively. To date, no genomic biomarkers are clinically applied that may aid in identifying high-risk patients.

Here, we combined existing approaches and technologies (FAIRE-seq, ChIP-seq, expression analyses, and survival data) as a potential “genomics pipeline” for biomarker discovery. FAIRE-seq analyses in prostate cancer specimens led to the identification of a large set of transcription factor motifs, including AR and multiple of its interaction partners. As a proof-of-principle, we studied the genomic behavior of AR. General applicability of such a genomic pipeline remains to be determined, and future studies should be aimed at assessing genomic features of other hits from the motif analyses to illustrate whether this approach is also applicable for other factors apart from AR.

We illustrate that prognostic biomarkers for the survival of patients with prostate cancer can be identified through the assessment of AR/chromatin interaction landscapes in tumor samples. By comparing AR binding patterns in primary tumor tissue specimens with those found in tumors that have an acquired resistance to treatment, a reprogramming of the AR interactome was observed. This reprogramming has far-reaching consequences, in which a distinct and unique gene set with acquired AR-responsive features provides prognostic potential for the survival of patients with prostate cancer, independent of classical prognostic parameters and clinical risk stratification system (D’Amico). Differential AR binding sites were clearly enriched for specific chromosomes, and a strong enrichment of resistance-associated AR binding sites was found at chromosome 8. Chromosome 8 has previously been implicated in prostate cancer progression, containing multiple oncogenes, including Myc (El Gammal *et al*, 2010). A selective enrichment of AR binding sites at distinct chromosomal regions could yield direct biologically relevant information on prostate cancer progression and may uncover drivers in ADT resistance.

Since the gene set identified in tumors with an acquired resistance yields prognostic potential in both cohorts of patients with primary prostate cancer, these data indicate that the AR-driven processes that were identified in the samples with an acquired resistance may already be active in the primary tumor, driving prostate tumor progression and metastasis formation. The expression of the acquired-resistance classifier in primary lesions may be used to identify patients with high risk of relapse after radical prostatectomy. Identification of high-risk patients is of clear clinical benefit, since it would enable closer monitoring of these patients. Since our classifier is based on differences between primary and resistant tumors, it remains to be determined whether these high-risk patients would be likely to respond to ADT or would be better treated with nonhormonal therapeutics.

AR is generally considered as the main driver in prostate cancer tumorigenesis and tumor progression. This notion is also further emphasized by the fact that all targeted therapies in prostate cancer treatment are directly aimed in a functional inhibition of AR activity. Nonetheless, analogous to recent finding in breast cancer (Robinson *et al*, 2011; Mohammed *et al*, 2015), other transcriptional regulators could potentially play a role in prostate cancer development and progression. AR ChIP-seq differential binding analysis yielded HOXB13 motif as selectively enriched in resistant tissue, suggesting a role of HOXB13 in resistant prostate cancer as found by others (Jeong *et al*, 2012). In addition, motif analyses on accessible chromatin regions by FAIRE-seq identified motifs for a large range of other transcription factors previously linked to prostate cancer development and progression, including CTCF, SP1, FOS, and ETS domain family of transcription factors (Shemshedini *et al*, 1991; Lu *et al*, 2000; Taslim *et al*, 2012; Chen *et al*, 2013). A subset of these transcription factors were previously defined as “druggable,” including ETS family members ETV1 (Rahim *et al*, 2014) and ERG (Nhili *et al*, 2013) as well as specific protein (SP) transcription factors (Safe *et al*, 2014). Out of the entire nine-gene signature, the three transcription factors (XBP1, ZBTB20, and KLF9) are previously described in relation to prostate cancer, while the other genes are not. KLF9 has been suggested to play a role in the progression of prostate cancer to a castration-resistant stage (Shen *et al*, 2014). XBP1 on the other hand is highly expressed in primary tumor, while hormone-refractory tumors show weak XBP1 expression (Takahashi *et al*, 2002; Cuperlovic-Culf *et al*, 2010). Furthermore, XBP1 is represented in the 150-core gene set of Sharma *et al*, while ZBTB20 is found in the gene set of Chen *et al*, and both signatures were based on AR binding in prostate specimens (Sharma *et al*, 2013; Chen *et al*, 2015). All these proteins may be of particular interest for new treatment options in prostate cancer, and future studies are aimed to further elucidate the potential roles of these transcription factors in prostate carcinogenesis as well as their therapeutic potential in this setting.

In summary, using integrative genomics of FAIRE-seq, AR ChIP-seq, publically available transcriptomic data and patient survival data, we successfully determined a minimal gene signature for the outcome of patients with prostate cancer. Even though the prognostic potential of these genes is apparent, future clinical trials should determine whether these genes could be informative for selective response to treatment of prostate cancer.

## Materials and Methods

### Clinical samples

Fresh frozen postoperative prostate needle biopsies from normal (pathologically validated tumor-free region of the peripheral zone) and tumor samples were obtained from prostatectomy specimens at the Netherlands Cancer Institute (Amsterdam, The Netherlands). The androgen-blockade-resistant tumor samples [transurethral resection of the prostate (TURP)] and lymph node metastases were obtained from the Erasmus University Medical Center (Rotterdam, The Netherlands). The hematoxylin and eosin (H&E) slides of all these cases were reviewed by our pathologists. See Appendix Table S1 for clinicopathological parameters. This study was performed in accordance with the Code of Conduct of the Federation of Medical Scientific Societies in the Netherlands and has been approved by the local medical ethics committees.

### Formaldehyde-assisted isolation of regulatory elements

FAIRE experiments were performed as previously described (Giresi & Lieb, 2009). Briefly, fresh frozen tissues were cross-linked with 1% formaldehyde for 20 min. Cells were washed, and nuclei were isolated as described (Zwart *et al*, 2013). Subsequently, chromatin was sonicated and cleared by centrifugation. The soluble chromatin was subjected to three consecutive phenol–chloroform–isoamyl alcohol (25:24:1) extractions. The samples were reverse-cross-linked overnight at 65°C and purified by ethanol precipitation. Purified samples were treated with RNase A and proteinase K and repurified by PCR purification kit (Roche).

### RNA isolation and mRNA expression

Total RNA was isolated after treatment with RNA-Bee reagent (Tel-Test, Inc.), and cDNA was synthesized from 500 ng RNA using SuperScript III Reverse Transcriptase (Invitrogen) with random hexamer primers. qPCR was performed with SYBR Green (GC Biotech) on a Roche LightCycler. Primer sequences for mRNA expression analysis are listed in Appendix Table S13.

### Chromatin immunoprecipitation (ChIP)

Chromatin immunoprecipitations were performed as described before (Jansen *et al*, 2013; Zwart *et al*, 2013). A total of 10 μg (ChIP-seq) or 5 μg (ChIP–qPCR) of antibody was used, with 100 μl (ChIP-seq) or 50 μl (ChIP–qPCR) of Protein A magnetic beads (Invitrogen). Antibodies used were AR-N20 (sc-618; Santa Cruz), CTCF (07-729; Millipore), and ERG (sc-353; Santa Cruz). Primer sequences for qPCR analysis are in Appendix Table S13.

### Solexa sequencing and enrichment analysis

DNA was amplified as described (Jansen *et al*, 2013). Sequences were generated by the Illumina HiSeq 2000 Genome Analyzer (using 50-bp reads) and aligned to the Human Reference Genome (assembly hg19, February 2009). Reads were filtered based on MAPQ quality: Only reads with MAPQ above 20 were considered to eliminate reads from repetitive elements. Enrichment over input control was determined using DFilter (Kumar *et al*, 2013) and MACS peak caller version 1.4 (Zhang *et al*, 2008). Only peaks called by both algorithms were used for the analysis. MACS was run with the default parameters, except *P* = 10^−7^ for ChIP-seq data. DFilter was run with bs = 100, ks = 50 for FAIRE data and bs = 50, ks = 30, refine, nonzero for ChIP data. Read counts, number of aligned reads, and number of peaks are shown in Appendix Tables S2 (FAIRE) and S4 (ChIP).

The raw and processed data are deposited in the Gene Expression Omnibus database (http://www.ncbi.nlm.nih.gov/geo/; accession No. GSE65478).

### Data analysis

Motif analyses were performed using Cistrome (cistrome.org), applying the SeqPos motif tool with default settings (region width: 600 bp; *P*-value cutoff: 0.001). Genomic distributions of binding sites were analyzed using the *cis*-regulatory element annotation system (CEAS). Differential binding analysis (DBA) was performed as described (Ross-Innes *et al*, 2012), considering all peaks that were found in at least three specimens, without control read subtraction and using a false discovery rate (FDR) below < 0.10. For integration with gene expression data, binding events were considered proximal to the gene when identified in a gene body or within 20 kb upstream from the transcription start site. Publically available ChIP-seq data for a number of factors in prostate cancer cell lines were used, and details are summarized in Appendix Table S12.

### Expression analyses and survival data

Time-series gene expression was analyzed using Short Time-series Expression Miner (STEM) (Ernst & Bar-Joseph, 2006) to identify gene sets that show significant temporal expression profiles. Default parameters were used, except for the minimum expression change set to 0.5 (based on the difference from 0). Two datasets of gene expression in LNCaP prostate cancer cells were used: GSE18684 and GSE8702. Details of the publically available gene expression datasets that were used in this study (GSE21034, GSE3933, GSE35988, GSE3325, GSE32269, GSE29079, GSE41408, GSE6811, GSE28680, GSE21887) are summarized in Appendix Table S12. These datasets were selected based on the availability of gene expression data from frozen tissue, relatively large sample sizes, measured on comprehensive gene expression platforms and clearly defined clinical groups. Clinical parameters (where available) of the gene expression datasets are summarized in Appendix Table S7. Cox regression regularized by an elastic net penalty from the glmnet package in R (Friedman *et al*, 2010) was used in order to select the optimal set of genes that are significantly associated with survival. Average centered probe levels were used. Double cross-validation was performed to assess the overall predictive ability of the procedure. In the outer loop, leave-one-out cross-validation was used. In the inner loop, regularization parameter lambda of the elastic net and the set of active covariates were determined with 10-fold cross-validation. Then, Cox regression was fitted with the selected variables and prognostic index estimated for the left-out sample. The final set of covariates for constructing Cox model was selected based on the average lambda value. In the validation cohort, prognostic index for each patient was defined as a sum of gene expression values multiplied by their corresponding coefficients derived from the Cox regression. Patients with prognostic index below or equal to zero were assigned to the low-risk group, while patients with the prognostic index above zero were assigned to the high-risk group. Differences in survival were assessed using log-rank test. Prognostic index was also used as a covariate in multivariate Cox regression analysis. The clinical parameters used in the multivariate analyses included pathologic Gleason score, T stage, lymph node status, and pretreatment PSA.

## For more information

http://www.nki.nl/divisions/molecular-pathology/zwart-w-group/

## Supporting Information

**Appendix**

**Expanded View Figures PDF**

**Table EV1**

**Table EV2**

**Table EV3**

**Table EV4**

**Table EV5**

**Review Process File**
