# Supplementary material for: Androgen receptor profiling predicts prostate cancer outcome
Source: EMBO Mol Med. 2015 Sep 27;7(11):1450–64. doi: 10.15252/emmm.201505424 (PMC4644377; doi:10.15252/emmm.201505424)
Supplement: Supplementary file 1 [file emmm0007-1450-sd1.pdf]

Appendix

Table of Contents

Appendix Figure Legends S1-S10

Appendix Figures S1-S10

Appendix Table Legends S1-S13

Appendix Tables S1-S13

Appendix Figure S1: Average read count of FAIRE-seq signal around TSS

Average profiles of the FAIRE-seq signal in tumor samples around the TSS of the genes with FAIRE-seq signal in the promoters.

Appendix Figure S2: Motifs enrichment scores and occupancy by the corresponding transcription factors

Scatter plot of the motif enrichment scores (-$\log_{10}$(p-value)) and ReMap overlap scores (-$\log_{10}$(e-value)) for the proteins corresponding to the motifs found in FAIRE-seq data from primary prostate tumors.

Appendix Figure S3: Ingenuity Pathway Analysis based on the motifs found in the FAIRE-seq regions enriched in primary tumor compared to normal tissue

Top canonical pathways, upstream regulators and networks identified are shown. For the networks, genes previously described to be involved in prostate cancer are highlighted in blue.

Appendix Figure S4: Biological replicate AR ChIP - seq

A. Venn diagram showing overlap of AR binding sites between two replicates.
B. Correlation of the read counts in the identified peaks in the two replicates.

Appendix Figure S5: Permutation testing in differential binding analysis

Scatter plot depicting the number of significantly differential peaks found in the permuted group labels, depending on the correlation between the permuted and original group labels. The highest number of peaks is found only when the permuted labels are equal to the original ones.

Appendix Figure S6: Background corrected AR ChIP signal

Averaged read count in peaks enriched in treatment resistant tumors (red) after the removal of the background due to possible amplification in chromosome 8 in one of the samples. Data are centered at AR peaks, depicting a 2.5 kb window around the peak. Read count is shown on the y-axis.

Appendix Figure S7: Enrichment score of the motifs found in ChIP-seq data

Heatmap illustrating enrichment of transcription factor motifs found enriched in ChIP-seq peaks present in either all the tumors, or differentially occupied in different clinical groups (primary or therapy resistant).

Appendix Figure S8: The presence of multiple AR binding sites at the KLK3 gene locus

ChIP-seq profile for AR in primary prostate tumor (green) and LNCaP cells (blue, GSM916521) showing a snapshot of AR binding sites within 20kb upstream of the transcriptional start site of KLK3.

Appendix Figure S9: Heatmap of gene expression of gene set I and III in hormone deprived LNCaP cells (GSE8702)

Heatmap illustrating gene expression of androgen responsive genes from geneset I and geneset III (Table EV5) in hormone deprived LNCaP cells (GSE8702).

Appendix Figure S10: Validation of the gene expression classifier in publicly available cohorts

Validation of the gene expression classifier (AMOTL1, DNER, EXT2, HSD17B14, KLF9, PMFBP1, RBM33, XBP1 and ZBTB20) in a number of gene expression cohorts with tissue samples at different stages of tumor development and progression (benign (normal), primary and metastatic tissue) available. Details of the clinical gene expression datasets are summarized in Appendix Table S12.

Appendix Figure S1

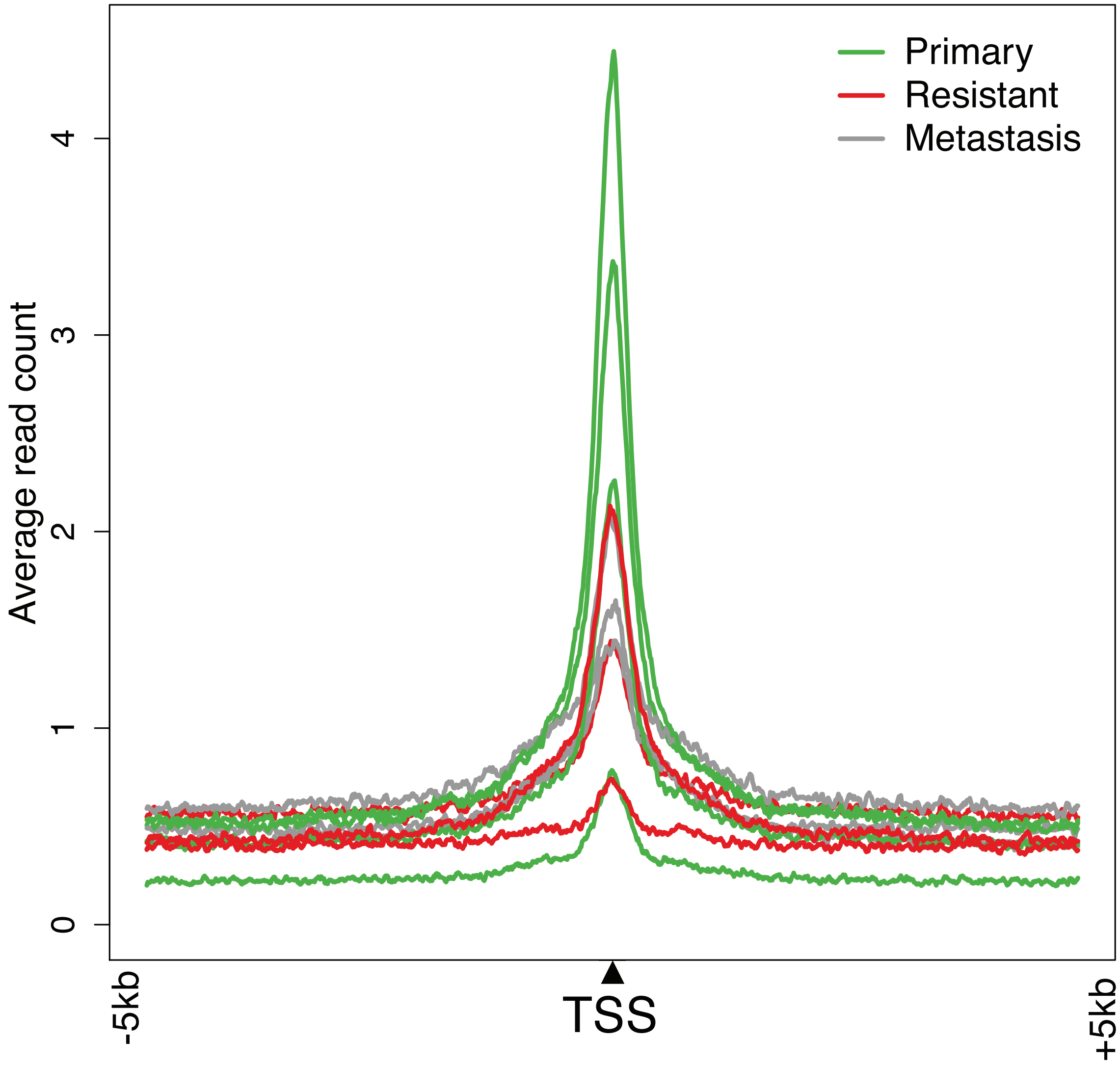

Appendix Figure S2

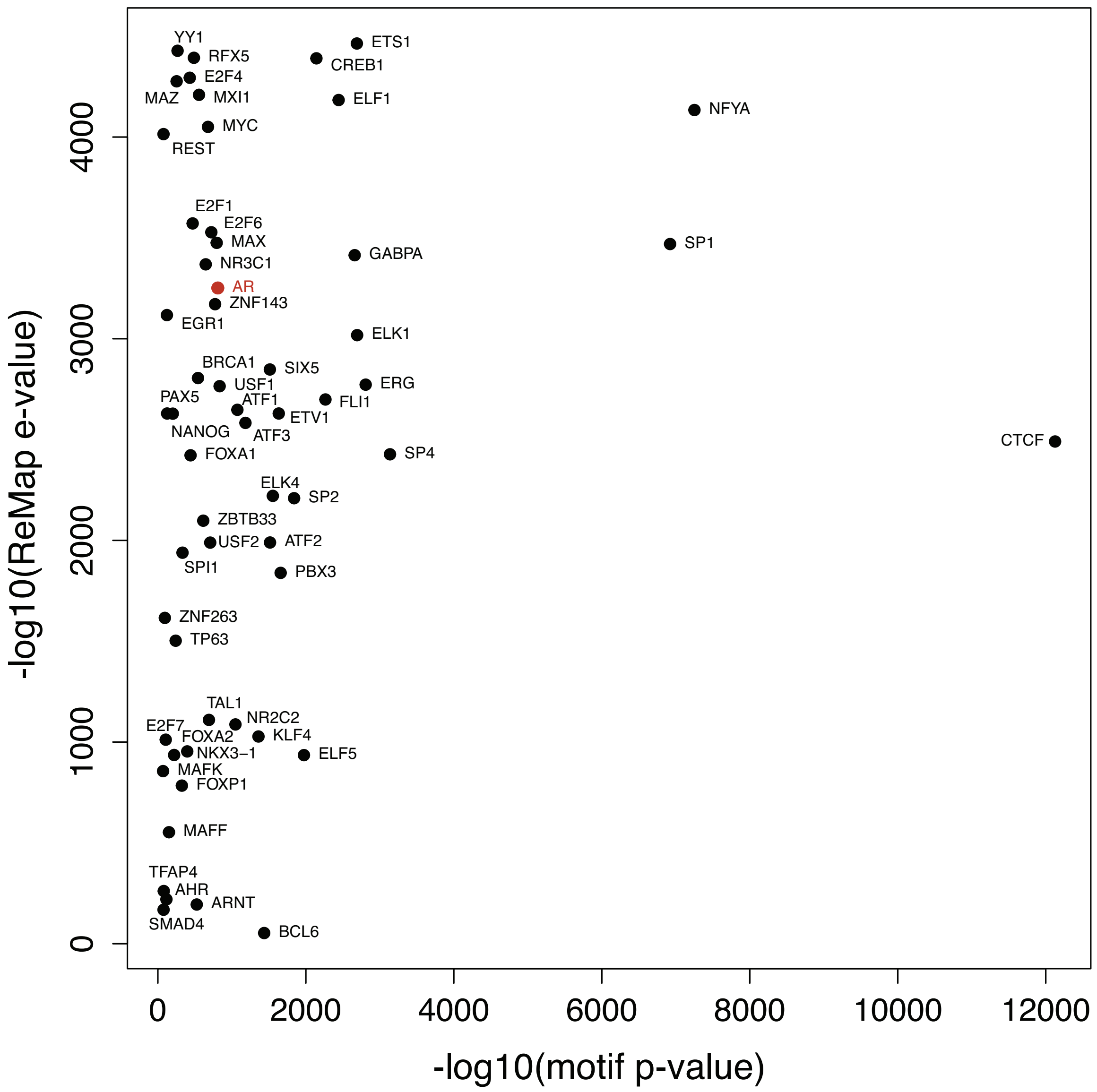

Appendix Figure S3

A

Top Canonical Pathways
ERK/MAPK Signaling
Telomerase Signaling
Estrogen-mediated S-phase Entry
Cell Cycle: G1/S Checkpoint Regulation
HGF Signaling

Top Upstream Regulators
EGFR
NOTCH1
ZBTB10
FOXA1
IRF4

B

Gene Expression, Cell Cycle, Cell Death and Survival

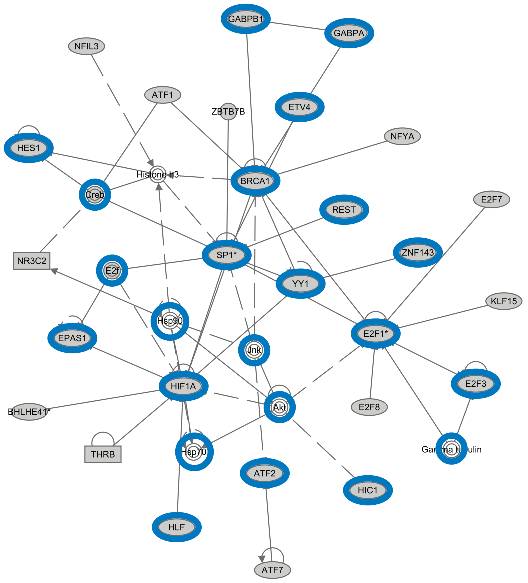

Gene Expression, Cell Cycle, Hepatic System Development and Function

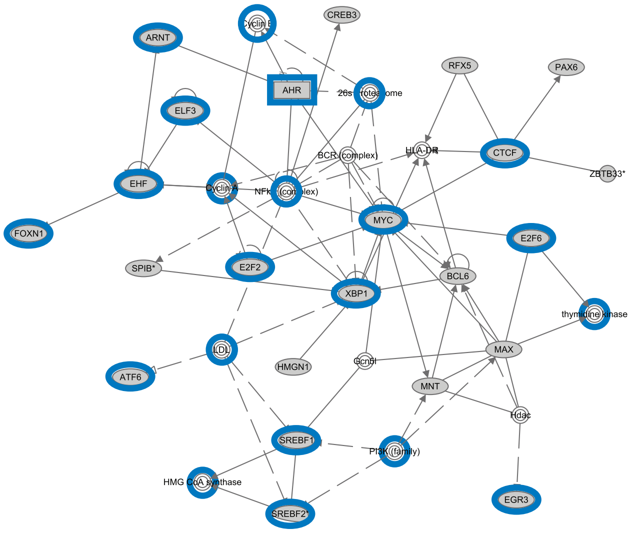

Appendix Figure S4

A

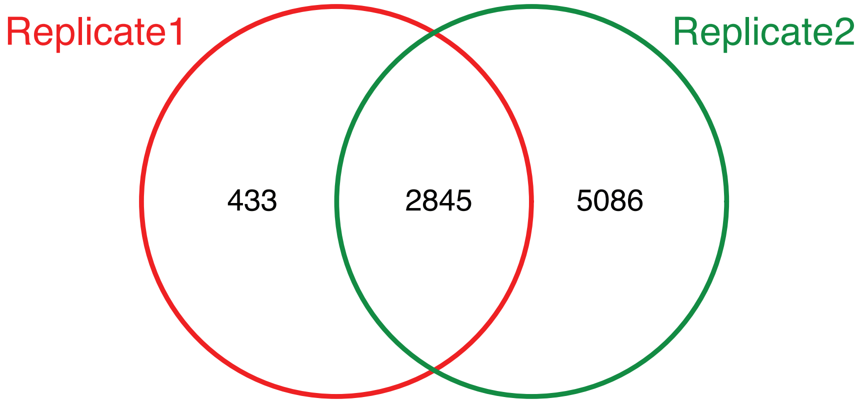

B

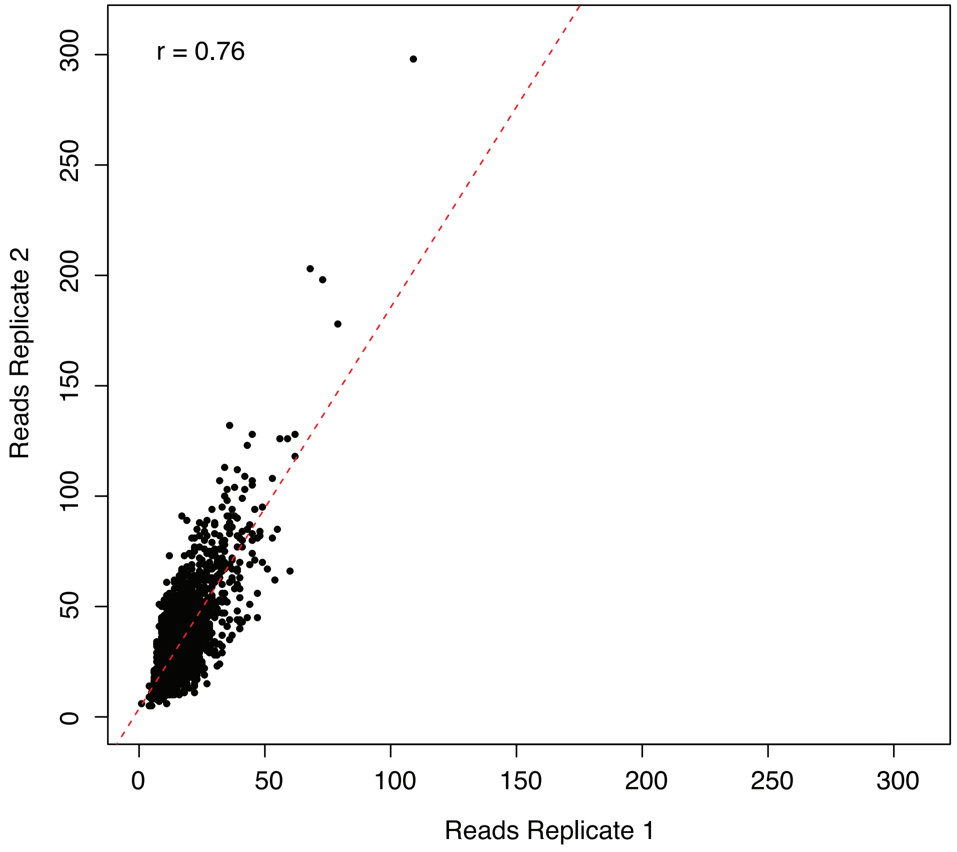

Appendix Figure S5

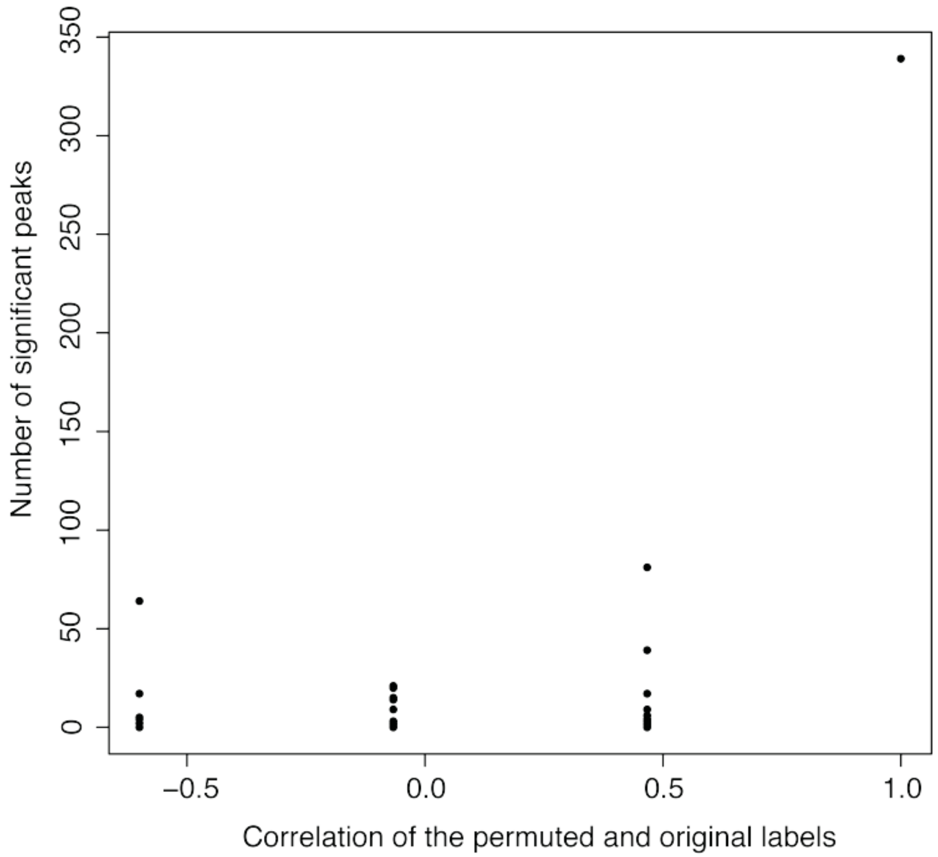

Appendix Figure S6

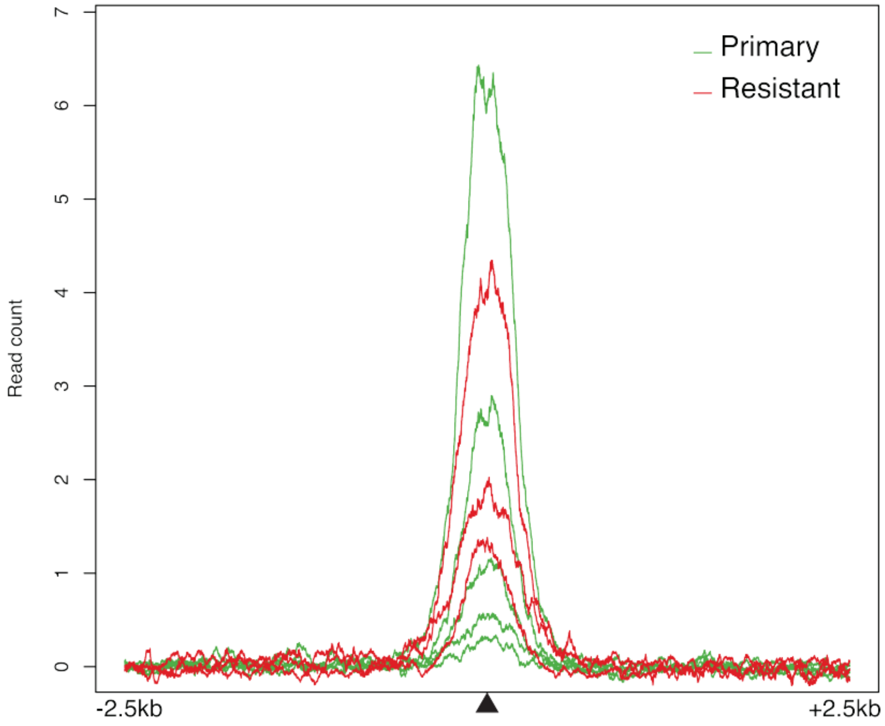

Appendix Figure S7

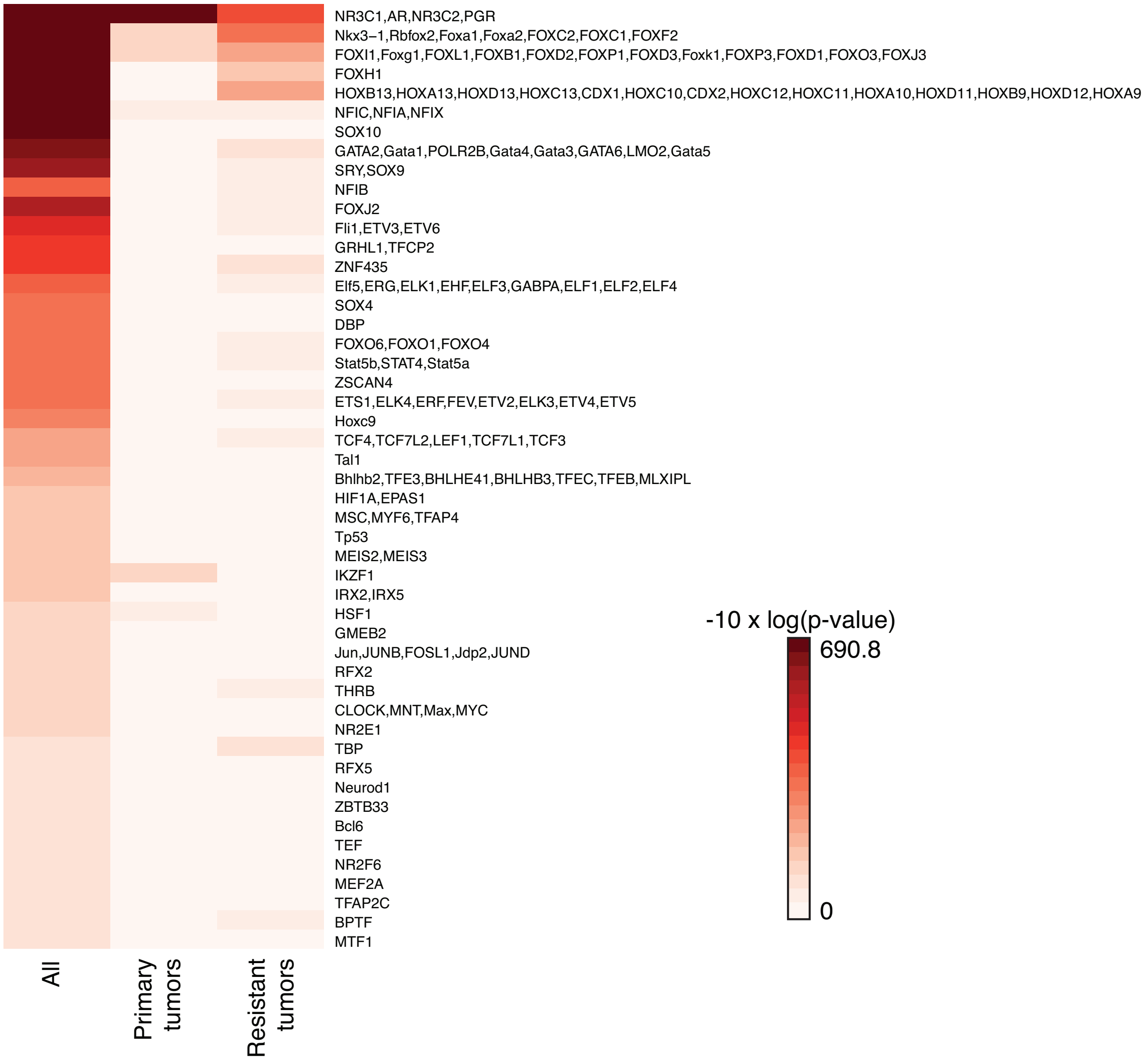

Appendix Figure S8

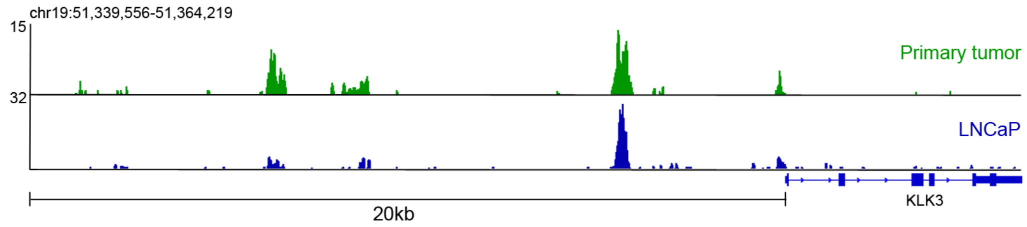

Appendix Figure S9

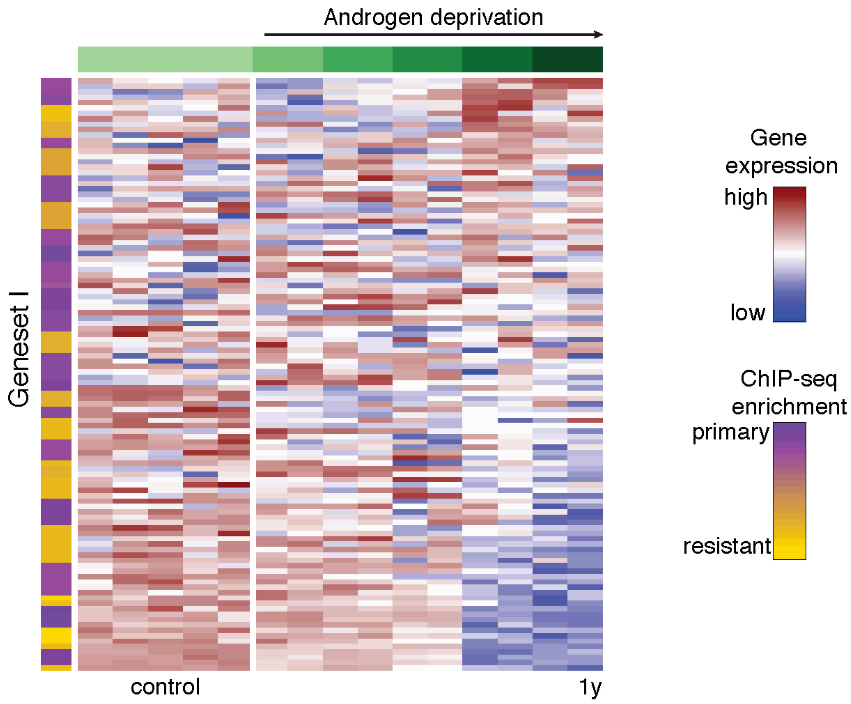

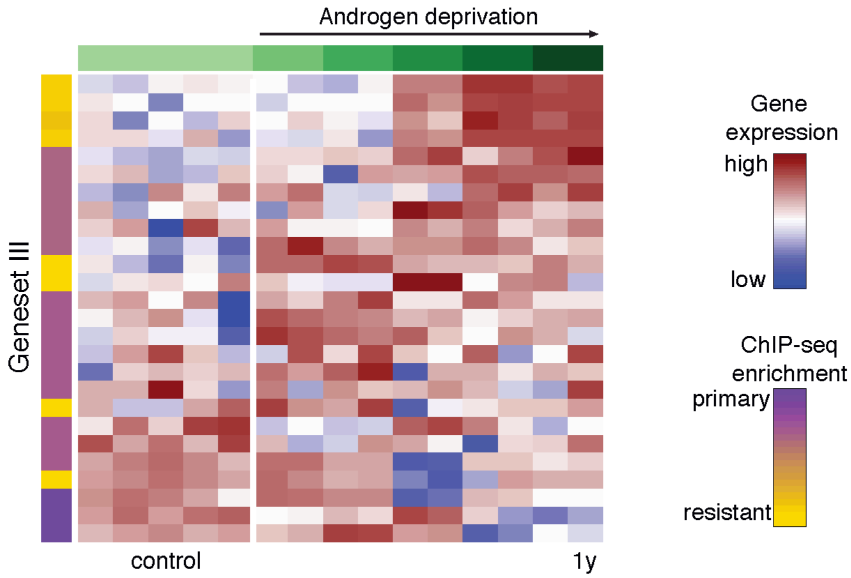

Appendix Figure S10

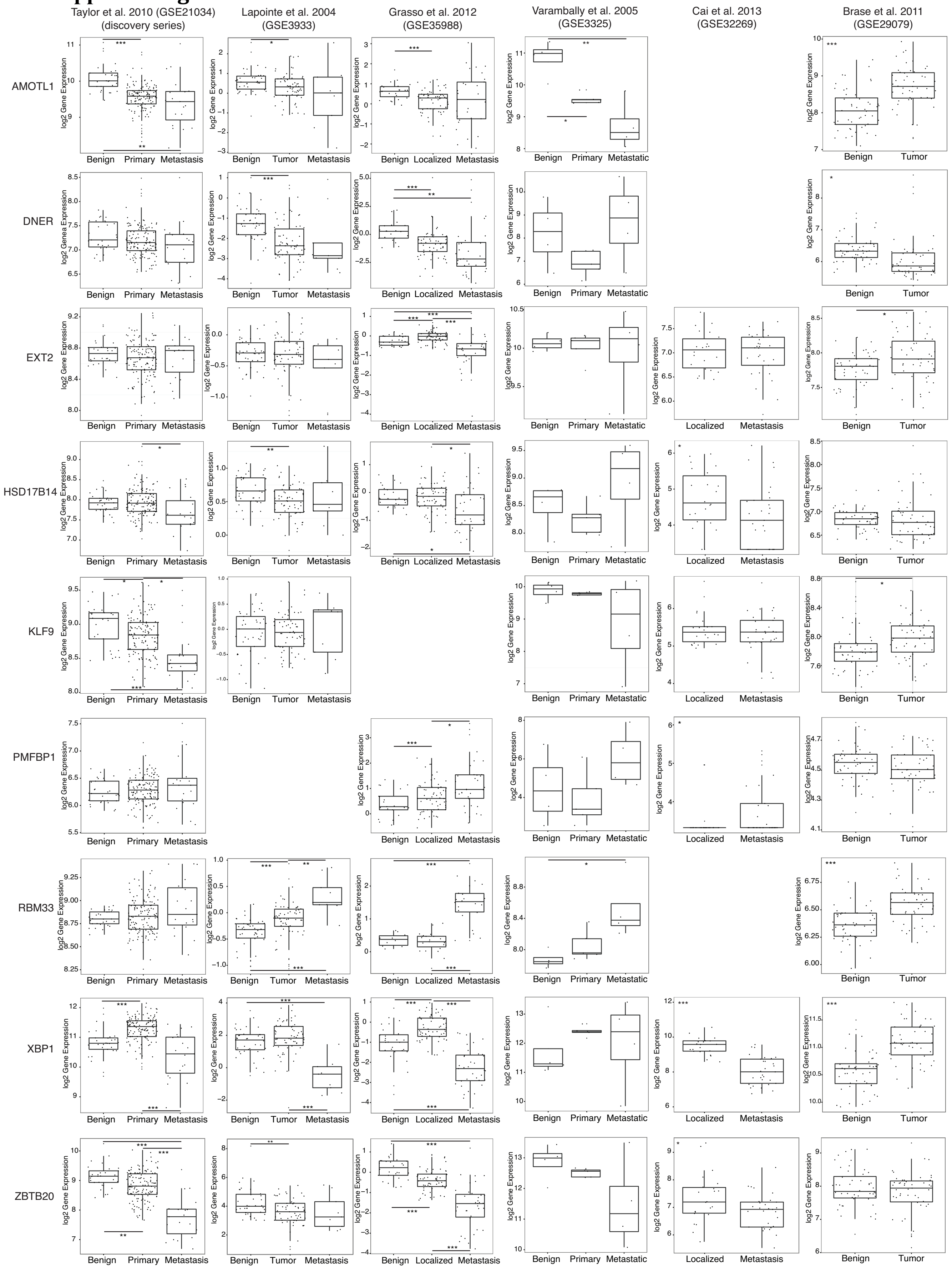

Appendix Table Legends

Appendix Table S1: Summary of the clinicopathological parameters

Appendix Table S2: Illumina sequencing data for FAIRE-seq, number of sequenced and aligned reads and number of peaks

Appendix Table S3: Top ten motifs (where available) found in FAIRE-seq peaks in individual patients

Appendix Table S4: Illumina sequencing data for ChIP-seq, number of sequenced and aligned reads and number of peaks

Appendix Table S5: Top transcription factors that overlap the regulatory regions associated with the 9 prognostic genes

Appendix Table S6: Cox regression coefficients for the genes constituting 9-gene prognostic signature.

Appendix Table S7: Clinical characteristics of the public patients cohorts used in the study

Appendix Table S8: Multivariate Cox regression with 9-gene based prognostic index and clinical parameters used as covariates

Appendix Table S9: AUC values for classification of patients who did or did not develop biochemical recurrence within 5 years after treatment by 9-gene prognostic signature, clinical parameters only and combined classifier

Appendix Table S10: Comparison of patients stratification in Taylor et al. cohort by D'Amico risk assessment and 9-gene classifications

Appendix Table S11: Hazard ratios and p-values for 9-gene classifier and D'Amico risk groups

Appendix Table S12: Details of the publically available ChIP-seq and clinical gene expression datasets

Appendix Table S13: qPCR primer sequences

Appendix Table S1

Sample	#	Experiment	Gleason score	PSA (ng/ml)	Therapy	Stromal/ epithelial ratio
Normal						
	1	FAIRE	4+5= 9	10	-	1.5
	2	FAIRE	3+4=7	13	-	1
	3	FAIRE	3+3=6	5.3	-	2.3
	4	FAIRE	4+3=7	6.6	Bicalutamide/Cyproteron Acetate	1
Primary tumors						
	1	FAIRE/AR ChIP	3+4=7	15.65	-	
	2	FAIRE/AR ChIP	3+4=7	16.28	-	
	3	FAIRE/AR ChIP	4+5=9	8.5	-	
	4	FAIRE/AR ChIP	4+5=9	38	-	
	5	AR ChIP qPCR	4+4=8	6.8	-	
	6	AR ChIP qPCR	4+5=9	51	Bicalutamide	
	7	AR ChIP qPCR	4+5=9	7.7	-	
	8	AR ChIP qPCR	4+4=8	7	-	
	9	ERG/CTCF ChIP/mRNA expression	4+3=7	11.2	-	
	10	ERG/CTCF ChIP/mRNA expression	4+4=8	8.2	Dutasteride	
	11	ERG/CTCF ChIP/mRNA expression	3+4=7	15.5	-	
	12	mRNA expression	4+3=7	7.5	Bicalutamide	
Resistant tumors						
	1	AR ChIP	5+5=10	6.5	Bicalutamide/LHRH analogue	
	2	FAIRE/ AR ChIP	5+5=10	511	Cyproteron Acetate+LHRH analogue	
	3	FAIRE/ AR ChIP	5+5=10	35	LHRH analogue	
	4	FAIRE/AR ChIP qPCR	3+5=8	45.6	LHRH analogue	
	5	AR ChIP qPCR	3+3=6	4	LHRH analogue	
	6	AR ChIP qPCR	4+3=7	102	LHRH analogue	
	7	AR ChIP qPCR	4+3=7	23	LHRH analogue	
	8	AR ChIP qPCR	4+4=8	59	LHRH analogue	
Metastasis						
	1	FAIRE	5+5=10	NA	-	
	2	FAIRE	4+4=8	254	LHRH analogue/Cyproteron Acetate	
	3	FAIRE	5+5=10	17	-	

Appendix Table S2

SampleID	Type	Total number of reads	Mapped reads	% mapped reads	Reads after MAPQ filtering	No Peaks	Average peak width
wz359	Turp	37452682	35655681	95.2	28518525	80	285
wz360	Naive	36827373	34441896	93.52	26405791	6450	272
wz361	LN	39562972	37594752	95.03	29920081	2043	308
wz362	LN	29130845	27291106	93.68	21657931	281	217
wz363	LN	27253810	25789354	94.63	20303729	1313	205
wz364	Naive	18306926	17002416	92.87	12569947	1579	154
wz365	Naive	32197589	30568523	94.94	24233246	13348	278
wz366	Naive	28992853	27590961	95.16	21709350	2243	231
wz367	Turp	28372546	26836918	94.59	20611332	3497	218
wz368	Turp	27545618	26061843	94.61	20534960	5754	429
wz369	Normal	19147127	17986187	93.94	15499657	50	157
wz371	Normal	21599945	19883501	92.05	17192503	472	200
wz382	Normal	26080719	25043481	96.02	21893547	61	177
wz383	Normal	23167347	22177458	95.73	19302469	2837	272

Appendix Table S3

Primary tumors				Resistant tumors			Metastasis		
wz360	wz364	wz365	wz366	wz359	wz367	wz368	wz361	wz362	wz363
CTCF	CTCF	CTCF	SP1	Too few peaks	CTCF	TFAP2B	ATF6	SP1	SP1
SP1	SP1	SP1	CTCF		SP1	REST	JUNB	TFEB	CTCF
NFIC	FOXA1	NFIC	ETV4		SP4	ZIC1	ATF1	FOXN1	ERG
ETS1	NFIB	SP4	ATF6		ATF6	SP1	ATF2	PBX3	HMGN1
ERG	NFIX	ERG	ERG		ETV4	TAL1	XBP1	ETS1	ATF2
ETS2	HIC1	ETV4	ATF2		Zscan10	MYOG	GMEB2		Zscan10
ATF2	NEUROD1	SP4	NFIC		ETS1	UBP1	SP1		PBX3
ELK4	SP4	Zscan10	SP4		ERG	SREBF2	CTCF		ATF6
SP4	PBX3	SP2	PBX3		PBX3	WT1	IRF4		SP4
PBX3	RFX2	ETS1	ELF2		ATF1	PAX4	PAX2		ETS1

Appendix Table S4

ID	Type	Total number of reads	Mapped reads	% mapped reads	Reads after MAPQ filtering	No Peaks	Average peak width
wz228	Naive	9928626	7080840	71.31742096	5313577	3278	305
wz229	Naive	12243485	11160623	91.15560643	9537336	7932	322
wz251	Naive	13782549	12232556	88.7539453	10340965	754	209
wz252	Naive	18146927	16009388	88.22093129	13293804	402	209
wz253	Naive	13040014	11254994	86.31121102	9592740	17511	339
wz241	Turp	16518987	14727645	89.15586047	12495762	739	181
wz242	Turp	16382421	14441817	88.15435155	12315771	238	175
wz243	Turp	15621538	13967477	89.41166356	11952448	1779	271
wz255	Naive	23982305	22739491	94.81778753		input	
wz256	Naive	27642177	26387234	95.46004282		input	
wz247	Turp	28171838	26825849	95.22221802		input	

Appendix Table S5

Transcription Factor	Observed Overlap	Expected Overlap	log10(e-value)
TFAP2C	12	0.67065	11.031
BRD3	7	0.0953	10.885
NR3C1	9	0.30875	10.274
AR	11	0.63405	10.03
POU2F2	6	0.06915	9.844
FOXA1	12	1.05445	8.825
IRF3	3	0.0029	8.392
BRD2	7	0.2521	7.987
ERG	9	0.65905	7.446
MYC	8	0.56905	6.783

Appendix Table S6

Gene	Coefficient
DNER	0.008368
EXT2	0.000676
AMOTL1	0.002325
RBM33	-0.004953
ZBTB20	-0.004538
XBP1	-0.001385
PMFBP1	-0.035441
HSD17B14	-0.004357
KLF9	-0.002588

Appendix Table S7

Characteristic	Taylor et al. (GSE21034)		Boormans et al. (GSE41408)	Lapointe et al. (GSE3933)		Brase et al. (GSE29079)
	Primary	Metastatic	Primary	Primary	Metastatic	Primary
**Age**						
Median	58.2	59	62	59	61	65
Mean	58.3	60.2	61.8	59.2	60.7	63.6
Standard deviation	7	8.6	5.8	6.6	3.9	5.8
Min-Max	37.3 - 83.0	41 - 82	49 - 73	45 - 72	55 - 66	48 - 74
**Pre-treatment PSA (ng/ml)**				na		
Median	6	18.8	10.75			11.9
<4	27 (15%)	3 (8.1%)	4 (8.3%)			2 (4.3%)
4-10	118 (65.2%)	8 (21.6%)	19 (39.6%)			19 (40.4%)
>10	35 (19.3%)	21 (56.8%)	23 (47.9%)			25 (53.2%)
Not available	1 (0.5%)	5 (13.5%)	2 (4.2%)			1 (2.1%)
**Pathologic Gleason score**						
6	41	-	23	24	-	4
7	74	2	16	22	-	32
8	8	3	8	10	-	1
9	7	4	1	5	-	10
10	1	10	-	-	-	
Not available	-	-	-	1	9	-
**Pathologic stage**						
pT2	85 (65%)	1 (5.3%)	15 (31.25%)	29 (46.8%)	-	16 (34.0%)
pT3	40 (30.5%)	7 (36.8%)	21 (43.75%)	28(45.2%)	5 (55.6%)	29 (61.7%)
pT4	6 (4.5%)	2 (10.5%)	12 (25.0%)	-	1 (1.1%)	2 (4.3%)
Not available	-	9 (47.4%)	-	5 (8.0%)	3 (33.3%)	-
**Lymph Node Status**						
Negative	102	3	44	52	0	30
Positive	6	10	4	5	5	13
Not available	23	6	-	5	4	4

Appendix Table S8

Covariate	Taylor et al. HR (CI)	Taylor et al. p	Boormans et al. HR (CI)	Boormans et al. p
9-gene Signature Prognostic Index	2.10 (1.43-3.07)	1.00E-04	0.80 (0.37-1.71)	0.56
Gleason Score				
<7	reference		reference	
7	1.86 (0.40-8.66)	0.43	0.70 (0.06-8.30)	0.77
>7	5.69 (1.00-32.32)	0.05	13.01 (1.47-114.84)	0.02
T stage				
T2	reference		reference	
T3	1.58 (0.56-4.45)	0.39	0.74 (0.06-9.70)	0.81
T4	2.22 (0.46-10.66)	0.32	4.09 (0.34-49.31)	0.27
Lymph Node Status				
Negative	reference		reference	
Positive	3.97 (1.08-14.62)	0.04	3.23 (0.56-18.57)	0.19
Pre-treatment PSA				
<4	reference		reference	
4-10	0.72 (0.20-2.59)	0.61	0.06 (0.00-0.95)	0.05
>10	0.89 (0.24-3.32)	0.86	0.30 (0.04-2.15)	0.23

Appendix Table S9

Classifier	AUC	CI.low	CI.high
Clinical Parameters only	0.83	0.72	0.94
9-gene signature	0.86	0.77	0.96
9-gene signature + Clinical parameters	0.902	0.82	0.98

Appendix Table S10

		9-gene classifier	
		Low risk	High risk
D'Amico groups	Low risk	35	26
	Intermediate risk	23	20
	High risk	12	15

Appendix Table S11

	HR (CI)	p
**9-gene prognostic signature**		
Low-risk	reference	
High-risk	5.134 (2.20-13.04)	0.0006
**D'Amico prognostic group**		
Low-risk	reference	
Intermediate-risk	1.10 (0.91-3.04)	0.85
High-risk	3.85 (1.55-9.52)	0.004

Appendix Table S12

Dataset	ChIP factor	Cell line	Reference
GSM916521	AR	LNCaP	Jin, H.J. et al. (2014)."Cooperativity and equilibrium with FOXA1 define the androgen receptor transcriptional program." Nat Commun 5:3972
GSM916523	FOXA1	LNCaP	Jin, H.J. et al. (2014)."Cooperativity and equilibrium with FOXA1 define the androgen receptor transcriptional program." Nat Commun 5:3972
Encode	CTCF	LNCaP	http://genome.ucsc.edu/cgi-bin/hgTrackUi?db=hg19&g=wgEncodeOpenChromChip
GSM699633	NKX3-1	LNCaP	Tan, P.Y. (2012). "Integration of regulatory networks by NKX3-1 promotes androgen-dependent prostate cancer survival." Mol Cell Biol 32:399-414
GSM1145322	EVT1	LNCaP	Chen, Y. et al. (2013). ETS factors reprogram the androgen receptor cistrome and prime prostate tumorigenes in response to PTEN loss." Nat med 19:1023-9
GSM1193658	ERG	VCaP	Sharma, N.L. (2014). " The ETS family member GABPα modulates androgen receptor signalling and mediates an aggressive phenotype in prostate cancer." Nucleic Acids Res 42:6256-69
GSM699637	PolII	LNCaP	Tan, P.Y. (2012). "Integration of regulatory networks by NKX3-1 promotes androgen-dependent prostate cancer survival." Mol Cell Biol 32:399-414
GSM686928	H3K4me1	LNCaP	Wang, D. et al. (2011)." Reprogramming transcription by distinct classes of enhancers functionally defined by eRNA." Nature 474: 390-4
GSM503907	H3K4me3	LNCaP	He, H.H. et al. (2010). "Nucleosome dynamics define transcriptional enhancers." Nat Genet 42: 343-7
GSM1071288	AR	malignant prostate tumor 1 (M1) [ChIP-exo replicate 1]	Chen Z et al. (2015). "Agonist and antagonist switch DNA motifs recognized by human androgen receptor in prostate cancer." EMBO J 34(4):502-16
GSM1071290	AR	malignant prostate tumor 2 (M2) [ChIP-exo replicate 1]	Chen Z et al. (2015). "Agonist and antagonist switch DNA motifs recognized by human androgen receptor in prostate cancer." EMBO J 34(4):502-16
GSM1071292	AR	malignant prostate tumor 3 (M3) [ChIP-exo replicate 1]	Chen Z et al. (2015). "Agonist and antagonist switch DNA motifs recognized by human androgen receptor in prostate cancer." EMBO J 34(4):502-16
GSM1071294	AR	malignant prostate tumor 4 (M4) [ChIP-exo replicate 1]	Chen Z et al. (2015). "Agonist and antagonist switch DNA motifs recognized by human androgen receptor in prostate cancer." EMBO J 34(4):502-16

Dataset	Details	Reference
GSE21034	181 primary, 37 metastatic and 149 matched normal samples. Median of 5 years clinical follow-up.	Taylor, B. S. (2010). "Integrative genomic profiling of human prostate cancer." Cancer Cell 18(1): 11-22
GSE3325	13 individual benign prostate, primary and metastatic prostate cancer samples and 6 pooled samples from benign,primary or metastatic prostate cancer tissues	Varambally, S. (2005). "Integrative genomic and proteomic analysis of prostate cancer reveals signatures of metastatic progression." Cancer Cell 8(5): 393-406.

GSE32269	22 primary and 29 metastatic prostate cancer samples	Stanbrough, M. et al. (2006). "Increased expression of genes converting adrenal androgens to testosterone in androgen-independent prostate cancer." Cancer Res 66(5): 2815-2825
GSE29079	48 benign and 47 prostate tumor samples	Brase, J. C. (2011). "TMPRSS2-ERG -specific transcriptional modulation is associated with prostate cancer biomarkers and TGF-beta signaling." BMC Cancer 11: 507.
GSE3933	62 primary prostate tumors, as well as 41 benign prostate samples and 9 lymph node metastases	Lapointe, J. et al. (2004). "Gene expression profiling identifies clinically relevant subtypes of prostate cancer." Proc Natl Acad Sci U S A 101(3): 811-816.
GSE35988	28 matched benign prostate tissues, 59 localized prostate cancer and 35 metastatic prostate cancer	Grasso, C. S. et al. (2012). "The mutational landscape of lethal castration-resistant prostate cancer." Nature 487(7406): 239-243.
	79 primary prostate cancer samples (37 patients with recurrent and 42 patients with nonrecurrent disease)	Glinsky, G. V. et al. (2004). "Gene expression profiling predicts clinical outcome of prostate cancer." J Clin Invest 113(6): 913-923.
GSE41408	48 prostate cancer samples from radical prostatectomies	Boormans, J.L. et al. (2013). " Identification of TDRD1 as a direct target gene of EG in primary prostate cancer." Int J Cancer 133(2):335-45
GSE6811	10 hormone sensitive and 25 resistant tumors	Tamura, K. et al. (2007)." Molecular features of hormone-refractory prostate cancer cellsby genome-wide gene expression profiles." Cancer Res 67; 5117
GSE28680	4 untreated prostate cancer samples and 4 hormone refractory prostate cancer samples	Sharma, N.L. et al. (2013). " The androgen receptor induces a distinct transcriptional program in castration-resistant prostate cancer in man." Cancer cell 23(1): 35-47
GSE21887	4 androgen-dependent growth (AD), 4 castration-induced regression nadir (ND), and 4 castration-resistant regrowth (CR) samples	Terada, N. et al. (2010). " Identification of EP4 as a potential target for the treatment of castration-resistant prostate cancer using a novel xenograft model." Cancer Res 70(4):1606-15

Appendix Table S13

Treatment sensitive enrichment AR binding sites	5' - 3'	genomic region	length
FW_1	CCACCTGTTCTCCAAAAACC	chr2:230,377,404-230,377,600	197
RV_1	AATACGATGCTTGCCAGAGG		
FW_2	CCCTCTTTTGTTTGCAGAGC	chr13:24,776,452-24,776,625	174
RV_2	CAACACTGGCATCCAACCT		
FW_3	CCCATGATAAAGTCAGGGAGA	chr10:122,836,341-122,836,539	199
RV_3	GACAGTGCGAGGCACTCAG		
FW_4	CCGGGACAGTCAGTTCTGTT	chr16:88,111,052-88,111,208	175
RV_4	CCTGTGGCTGCAACTTTCTC		
FW_5	GCGCGGGAGAGAAAGAGTTA	chr6:35,699,687-35,699,856	170
RV_5	CTCTCCCTCCCCGGACTAC		
FW_6	CTGGCTCTTTCCACTCCAAC	chr19:3,429,760-3,429,951	192
RV_6	AATCTGCAAAGCTCCGAGAA		

Treatment resistant enrichment AR binding sites	5' - 3'	genomic region	length
FW_1	ACAATGCCTGGCACACAATA	chr3:110,331,037-110,331,224	188
RV_1	ATCCAGGAGAAGCTCCGAGT		
FW_2	CCACCAAATATCCATCCCTGT	chr7:29,270,374-29,270,578	205
RV_2	ATGCTTGCTGGCACCATAA		
FW_3	TGTGTTTGCTGAGGTGTGGT	chr8:102,619,944-102,620,138	195
RV_3	GGTGAGATGCCTTCCAAAGA		

FW_4	TTTGAGCTACACCAGGCATCT	chr8:66,413,034-66,413,248	215
RV_4	ACTGAATGTCGGGCAGACTC		
FW_5	ACTTTTCTCCAATCCCTTCAA	chr8:136,589,398-136,589,568	171
RV_5	AACCCAAACAACCTCACAGC		
FW_6	TGCCGAAGAAATGTAGTTGG	chr3:101,029,583-101,029,745	163
RV_6	TAGGAGGTGGGGAAACATTG		

CTCF binding sites	5' - 3'	genomic region	length
FW_CTCF1	TGGCAGCTCTGGGTTTAGAA	chr17:32,688,683-32,688,778	96
RV_CTCF1	CCCTAGAGAACAGAACCCCG		
FW_CTCF2	CTAGGCGGCTGGACAGAAGTG	chr1:109,806,454-109,806,552	99
RV_CTCF2	AACAGGTGGCAGCATTGACCT		
FW_CTCF3	TCGGCTCCACCAGTCTGT	chr1:114,889,237-114,889,341	104
RV_CTCF3	GCCCTCTAATGGACACTGCC		
FW_CTCF4	GGCCTTCTAGGTGCAGAGAC	chr12:53,374,787-53,374,902	116
RV_CTCF4	GATGGAGCAGCCGCCTATTA		
FW_CTCF5	TTAGTGTCTGGCATTTGCAGC	chr21:41,160,377-41,160,446	70
RV_CTCF5	CAGCAGGCGGGATGATAGTC		
FW_CTCF6	TTCCGAATTGGGGGAGGGTA	chr12:12,857,732-12,857,808	77
RV_CTCF6	ATAGTTAGGCGCTCTGTGGC		

ERG binding sites	5' - 3'	genomic region	length
FW_ERG1	CAGTGTGCTGTTCTCCGTCT	chr11:114,050,099-114,050,179	81
RV_ERG1	CAGACGCAGGGCATTTTACA		
FW_ERG2	AATGCCAGGGAAACGCATTG	chr16:73,024,189-73,024,274	86
RV_ERG2	TCTTGCAGCCTGAGACATGG		
FW_ERG3	AGTGTCTGGCAAGGTGTTGT	chr17:60,803,337-60,803,426	90
RV_ERG3	CGGGAGATTGTGTCCAGGTT		
FW_ERG4	AGGTTCACCCTGTGCTTTGT	chr19:46,246,446-46,246,498	53
RV_ERG4	AACCATAGTCCCTGGGTGTT		
FW_ERG5	ACTTGGAACAGCTAGACCTGC	chr21:31,588,540-31,588,669	130
RV_ERG5	AAGGGATCGGCTCATTTCTTGA		
FW_ERG6	TGTGTCTGCTAGTGAAGGGG	chr2:174,075,223-174,075,394	172
RV_ERG6	TGCATCATGTGACCTGGCTC		

AR ChIP & CTCF ChIP	5' - 3'	genomic region	length
negative control primer FW	CACACCCGCTCTACGATATGA	chr19:51,361,368-51,361,474	106
negative control primer RV	GAGCTCGGCAGGCTCTGA		

ERG ChIP	5' - 3'	genomic region	length
negative control primer FW	TGGCCCTTGATACTGGAGTC	chr11:69,573,441-69,573,595	155
negative control primer RV	GACATCCAAGGCAAGATGGT		

mRNA expression primers	5' - 3'	length
FW_CTCF	GGAGCCTGCCGTAGAAATTG	131
RV_CTCF	TAGCTGTTGGCTGGTTCTGTT	
FW_ERG	AGCATGCATTAACCGTGGAGA	115
RV_ERG	CAACGCCGACATCCTTCTCT	
FW_ETV1	TGCAGTCAAGAACAGCCCTT	76
RV_ETV1	GCTTCTGATCATAGGCACTGA	
FW_NKX3-1	CCGAGCCAGAAAGGCACTTG	86
RV_NKX3-1	CTTAGGGGTTTGGGGAAGCC	
FW_Androgen Receptor	AGAGTGCCCTATCCCAGTCC	97
RV_Androgen Receptor	CAGTCTCCAAACGCATGTCC	
FW_TBP	GTTCTGGGAAAATGGTGTGC	100
RV_TBP	GCTGGAAAACCCAACTTCTG	
FW_Beta-actin	CCTGGCACCCAGCACAAT	144
RV_Beta-actin	GGGCCGGACTCGTCATACT	
