# Supplementary material for: Androgen receptor profiling predicts prostate cancer outcome
Source: EMBO Mol Med. 2015 Sep 27;7(11):1450–64. doi: 10.15252/emmm.201505424 (PMC4644377; doi:10.15252/emmm.201505424)
Supplement: Supplementary file 2 [file emmm0007-1450-sd2.pdf]

Expanded View Figures

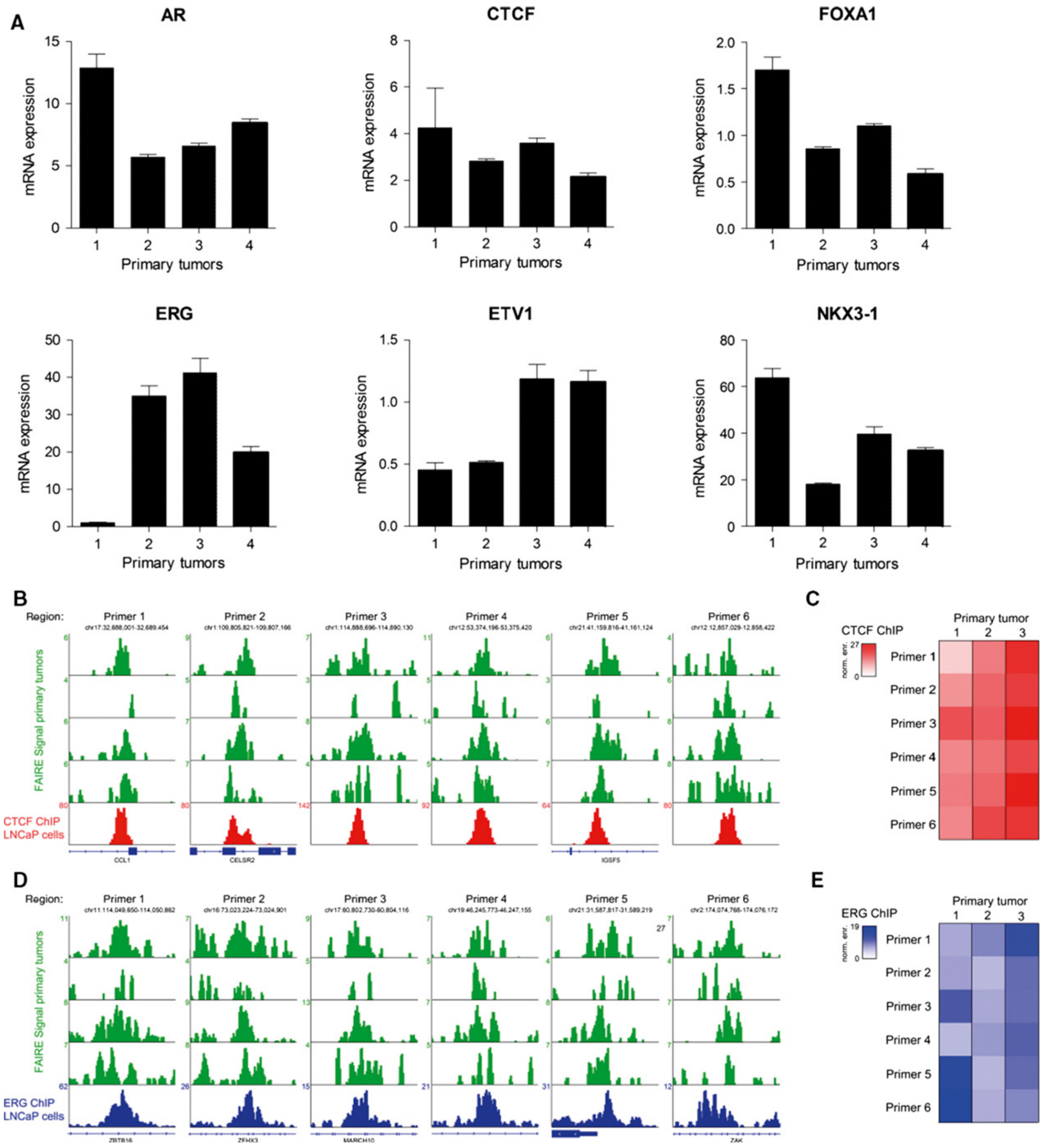

Figure EV1. Expression and occupancy of a set of transcription factors corresponding to the identified motifs at FAIRE peaks.

A Expression levels of AR, CTCF, FOXA1, ERG, ETV1, and NKX3-1 relative to TBP in four independent primary tumors. Error bars indicate SD from triplicate analysis.

B FAIRE-seq snapshots of accessible chromatin regions containing a CTCF motif in four primary tumors (green) and CTCF binding as assessed through ChIP-seq in LNCaP cells (red track, GSE33213).

C Heatmap, illustrating ChIP–qPCR-based enrichment of CTCF binding at accessible chromatin sites depicted in (B).

D FAIRE-seq snapshots of accessible chromatin regions containing an ERG motif in four primary tumors (green) and ERG binding as assessed through ChIP-seq in LNCaP cells (blue track, GSM1193658).

E Heatmap, illustrating ChIP–qPCR-based enrichment of ERG binding at accessible chromatin sites depicted in (D).

 © 2015 The Authors

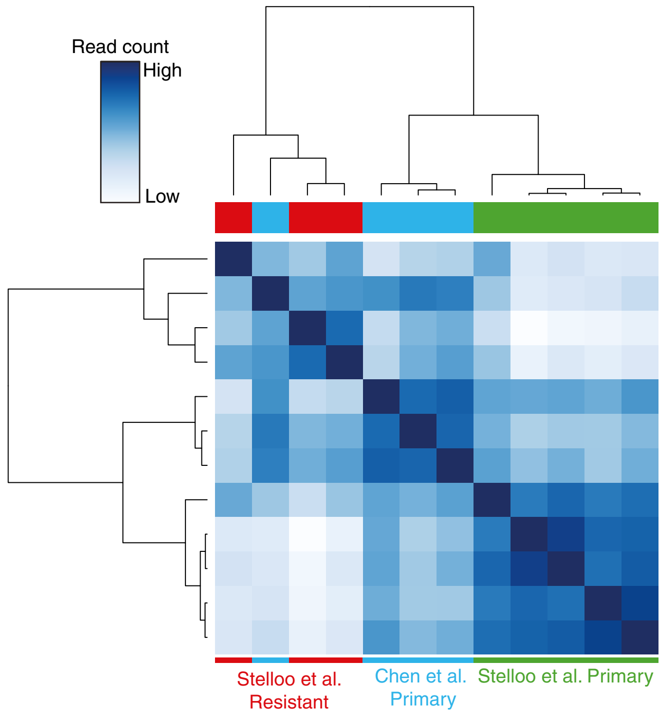

Figure EV2. Hierarchical clustering of Stelloo *et al*'s (this manuscript) and Chen *et al*'s data.
Heatmap of the correlation between the ChIP-seq data from Stelloo *et al* and Chen *et al* based on the 339 differential AR binding sites. Three out of four primary tumors from Chen *et al* cluster together with the primary tumors from this study.

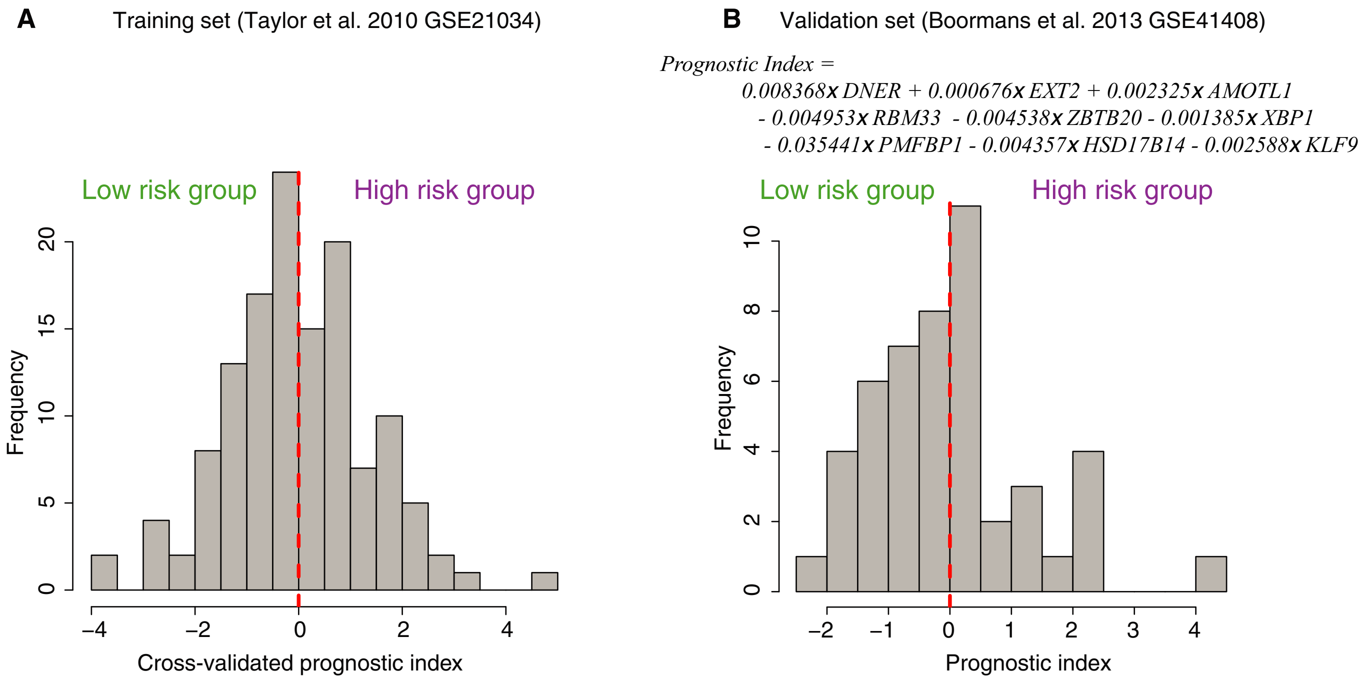

Figure EV3. Distribution of the prognostic indices in the training and validation sets.

A Histogram of the prognostic index distribution in the training dataset; patients were assigned to two risk groups based on their positive or negative prognostic index.

B Histogram of the prognostic index distribution in the validation dataset.

© 2015 The Authors

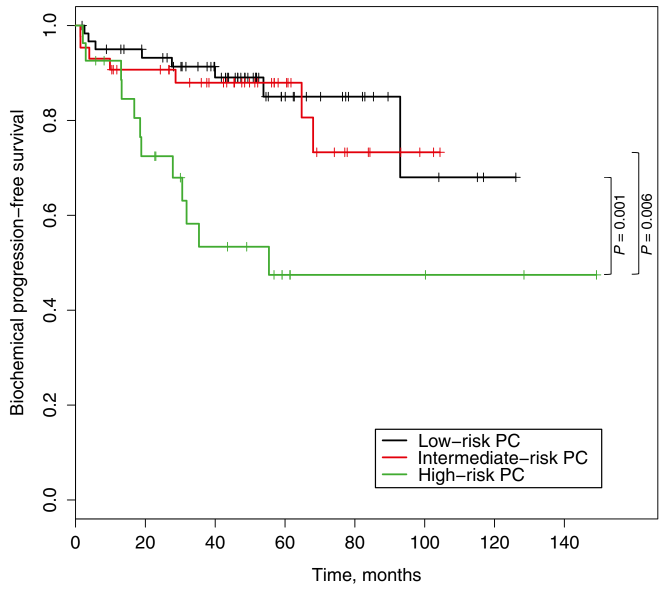

Figure EV4. Kaplan–Meier survival curves based on D'Amico classification.

Kaplan–Meier survival curves of patients from Taylor *et al* cohort stratified into three risk groups based on D'Amico classification using clinical parameters.

 © 2015 The Authors
