# Supplementary material for: Androgen receptor profiling predicts prostate cancer outcome
Source: EMBO Mol Med. 2015 Sep 27;7(11):1450–64. doi: 10.15252/emmm.201505424 (PMC4644377; doi:10.15252/emmm.201505424)
Supplement: Supplementary file 8 [file emmm0007-1450-sd8.pdf]

EMBO Molecular Medicine

Androgen Receptor profiling predicts prostate cancer outcome

Suzan Stelloo, Ekaterina Nevedomskaya, Henk G van der Poel, Jeroen de Jong, Geert JLH van Leenders, Guido Jenster, Lodewyk FA Wessels, Andries M Bergman and Wilbert Zwart

Corresponding author: Wilbert Zwart, Netherlands Cancer Institute

Review timeline:

Submission date:	01 May 2015
Editorial Decision:	15 June 2015
Revision received:	10 August 2015
Editorial Decision:	28 August 2015
Revision received:	01 September 2015
Accepted:	03 September 2015

Transaction Report:

(Note: With the exception of the correction of typographical or spelling errors that could be a source of ambiguity, letters and reports are not edited. The original formatting of letters and referee reports may not be reflected in this compilation.)

Editor: Roberto Buccione

1st Editorial Decision 15 June 2015

Thank you for the submission of your manuscript to EMBO Molecular Medicine. We are sorry that it has taken longer than usual to get back to you on your manuscript. In this case we experienced some difficulties in securing three appropriate reviewers and then obtaining their evaluations in a timely manner.

As you will see three Reviewers are globally quite positive, but do raise a number of issues, quite a few of which are fundamental. Although I will not dwell into much detail, I would like to highlight the main points.

Firstly, Reviewer 1 finds that the case for clinical utility was not made sufficiently clear and suggests a number of approaches to better define these aspects, which of course are of great importance for our title. This at the very least will require re-analysis, or possibly acquisition of new experimental data. The reviewer also asks for more details on patients, statistical tests, etc. We agree and to this effect, please note that all EMBO Press journals, including EMBO Molecular Medicine, now require a complete author checklist (http://embomolmed.embopress.org/authorguide#editorial3) to be submitted with all revised manuscripts. Provision of the author checklist is mandatory at revision stage; The checklist is designed to enhance and standardize reporting of key information in research papers and to support reanalysis and repetition of experiments by the community. The list covers key information for figure panels and captions and focuses on statistics, the reporting of reagents, animal models and human subject-derived data, as well as guidance to optimise data accessibility.

Reviewer 2 finds the manuscript to be disjointed in terms of the basic premise that it shows a

generally applicable integrative approach to identify cancer biomarkers. S/he is especially critical with respect to how the FAIRE data integrate with the other elements. We also agree that perhaps some further validation of the signature in the clinical sample would be of value and would increase the impact of the study. All in all, the comments are pertinent and require some action on your part in terms of re-writing and clarification of the issues raised, in addition to some limited additional experimentation.

Reviewer 3 offers a detailed list of points that should work quite well towards improving the impact and presentation of the data. I would also like to point out that this Reviewer also raises the issue of clinical relevance.

In conclusion, while publication of the paper cannot be considered at this stage, given the interest of your findings and the Reviewers' positive reception, we have decided to give you the opportunity to address the above concerns.

We are thus prepared to consider a substantially revised submission, with the understanding that the Reviewers' concerns must be addressed with additional experimental data where appropriate and that acceptance of the manuscript will entail a second round of review.

Please note that it is EMBO Molecular Medicine policy to allow a single round of revision only and that, therefore, acceptance or rejection of the manuscript will depend on the completeness of your responses included in the next, final version of the manuscript.

As you know, EMBO Molecular Medicine has a "scooping protection" policy, whereby similar findings that are published by others during review or revision are not a criterion for rejection. However, I do ask you to get in touch with us after three months if you have not completed your revision, to update us on the status. Please also contact us as soon as possible if similar work is published elsewhere.

I look forward to seeing a revised form of your manuscript as soon as possible.

***** Reviewer's comments *****

Referee #1 (Remarks):

The manuscript by Zwart et al identified a distinct Androgen Receptor/chromatin binding profile between primary and resistant prostate cancers which in turn may be prognostic. Such a signature could be very important in placing patients into groups that require differential treatment.

Overall the data are provocative and the chromatin binding studies completed well. There are a number of important classifications and further experiments that are required in order that the reader can fully agree with the author's conclusions.

Are the authors driving forward a test that can be used a priori or only after surgery. IF the former, then the "clinical" pre-treatment staging must be used in addition to the signature in a multivariate analysis. If the latter, then additional pathologic and post-operative factors must be used in such analyses. Finally, if the authors are predicting ADT response, then a cohort that did or did not have early PSA failure after ADT was initiated should be used. At present the rationale for the choice of cohorts and the clinical utility is unclear.

The details of the clinical biostatistical studies and KM analyses are very unclear. What are the clinical factors used in the multivariate analyses for post-prostatectomy patients for each cohort and are these independent of known post-prostatectomy factors (margins, percent tumour, GS, PSA, T category, etc)?. For each cohort tested there should be a table of patient characteristics and the nature of the test and the MV statistical results.

Does the score aid prognosis across all NCCN/D'Amico pre-treatment prognostic groups? The authors should test for this association.

The authors should complete a ROC-AUC curve for their signature to show utility above clinical variables alone.

Referee #2 (Comments on Novelty/Model System):

The authors have employed FAIRE-seq, ChIP-seq and transcript analysis to define patient tumour samples. ChIP-seq has been undertaken for the AR. The authors comment on the significant variability in both AR peaks and FAIRE sites from sample-to-sample. There is little benchmarking of the AR data to other tissue datasets in the paper (site overlaps). There is no commentary on the mean size of the FAIRE sites in each sample or how size variation and variation in the number of sites may impact on motif enrichment analysis. More detailed discussion and cross-comparison is needed given the heavy emphasis on an integrative approach.

Referee #2 (Remarks):

The authors have generated maps of accessible chromatin, AR binding sites and transcript profiles from patient tissue samples. They argue that this represents an generalizable integrative approach for identifying cancer biomarkers. It is not entirely clear how the FAIRE data inform the other elements of the paper. Presumably in the absence of these data the authors, could based on other studies, have generated AR ChIP-seq data and generated a biomarker signature by integrating the sites with transcript profiles and comparison with public databases.

A number of points need to be addressed to make this less disjointed and more convincing as a single unified piece of work:-

1. The authors specify that the majority of the FAIRE-seq sites are in promoters. However the reads distribution in figure 2F is centered at the peaks centres and not at e.g. TSSs. This potentially introduces a bias in their analysis.

2. From the legend of Fig 2H it is not clear which FAIRE-seq sites are taken into account? common?... only in one sample?

3. The AR is a well known driver of PC. Is there anything from their FAIRE-seq data that would show a major involvement of other TFs on target genes with a prognostic significance? For example MYC and other TFs seem to occupy also a central area in their nodes-based analysis. Are the motifs consistent across different individuals within the same clinical group?

4. Can the authors show overlaps of TFs binding with open regions? What is the overlap of AR binding sites with FAIRE sites as compared to others e.g. CTCF binding sites, ERG sites etc?

5. AR binding profiles differences were used to determine genes signature. Was the binding very heterogeneous compared to other papers? Were there any site overlaps with other published studies? Could differential AR binding along their gene signature be applied also for the AR binding profile in CRPC ARBSs in Sharma et al?

6. It would be useful to define the novel genes within the signature and to validate the the gene signature as a whole using qPCR in the clinical samples.

Finally in terms of the generalisability of the pipeline, it is clear that FAIRE-seq and ChIP-seq data can be compared to transcript profiles to find genes. However, for this approach to be generalizable the authors would need to show that the FAIRE approach is capable of identifying driving transcription factors in an unbiased manner, that ChIP-seq for those factors provides site maps that then enhance the filtering of differential transcripts to yield a more powerful biomarker signature. In this study at least as it is written it doesn't appear that FAIRE data alone has led the authors to the AR and it appears that the low and variable number of AR sites generated by ChIP-seq has restricted

the number of candidate genes within the signature. The manuscript needs carefully restructuring to clearly illustrate the power and value of all of the presented elements in concert.

Referee #3 (Remarks):

Summary

Stelloo and colleagues present a nice study of androgen receptor activity in four men with primary prostate cancer and three men with hormone relapsed disease, three with mets as well as some benign tissue. They use ChIP methods to identify binding sites in these different phases of the disease, including an approach called FAIRE-seq and attempt to demonstrate the distinct profiles. They construct a prognostic signature of nine genes and assess this in several public cohorts to demonstrate its clinical utility in predicting biochemical relapse.

Comments re Content
1. Abstract. It sounds in the abstract like all the data is taken from public datasets. Given that this is not the case (there are fresh ChIP studies of human material and cell lines) this should be made clear in the abstract. It fundamentally increases the power of the study and should right there to sell the story from the start!
2. Introduction. First para, second sentence. Patients are normally treated with "prostatectomy and/or radiotherapy" rather than the other way round.
3. Introduction. Fourth para, first sentence. This is not always the case. Men with recurrence are normally offered salvage radiotherapy after prostatectomy or locally advanced disease are often offered radiotherapy plus hormones or indeed prostatectomy and adjuvant RTx.
4. Introduction. Fourth para, 3rd sentence. Needs a reference. As do subsequent statements.
5. Introduction. Fourth para, final sentence. There are actually several papers that present "biomarkers" (signatures actually) that enable identification of prostate cancer patients at high risk of relapse (e.g. Ramos-Montoya et al EMM 2014 HES66/E2F1/AR signature, Irshad et al Nat Comms 2014 "indolence" signature, Lalonde et al Lancet Onc 2014 copy number signature).
6. Introduction. 5th para. 4th sentence. This comment about resistance to inhibitors needs a reference. (e.g. Antonarkis et al, NEJM).
7. Introduction. 5th para. Final sentence. There have been some nice reviews of these mechanisms of resistance which should probably be referenced (instead of individual studies). See Pienta et al Ca Cancer J Clin 2005 or most recently Lamb et al BJUI 2014.
8. Results. Figure 1. Ok
9. Results. Figure 2. Am confused by numbers here. Are there three or four primary? There are three in text. Four on figure 2C. Four on table S1.
10. Results. Figure 2. 2nd para page 6. This figure needs more numbers I think. What are the stats for the quantitative statements made here? A statement is made about there being an increase in chromatin accessibility in tumour samples but no numbers or tests are presented. Is this really true? There are only four samples.
11. Results. Figure 2. Panel B. These averaged wig file plots should all be on the same y axis. At the moment it is hard to compare the peak calls visually. In fact, it looks like there is no difference at all at the moment whereas, if the axis is changed, the difference will be more apparent (and hopefully send a more powerful message!). Also, why are these four representative peaks chosen?
12. Results. Figure 2. Panel C. needs stats.
13. Results. Figure 2. Panel E. There seems to be much more promoter binding in benign compared to tumour, which instead has much more intron and distal intergenic binding. I don't think this is commented on at all in the text.
14. Results. Figure 2. Panel G. What was the input list for IPA. Was it table S8? Either way, this needs to be made clear in the text.
15. Results. Figure 3. Panel B. Same as above.
16. Results. Figure 3. Panel C. I think this could be displayed more clearly with a subcluster plot. See Ramos Montoya et al, EMM 2014 Figure 2D for an example.
17. Results. Figure 3. Panel E. The key needs to be improved on here so that all the colours are specified. There seem to be more than two colours on each plot. Or if these are replicates then perhaps they should be averaged. See Ramos-Montoya et al, EMM 2014 Figure 2B for an example.

Figure 2A and C also gives an example of points mentioned above in 11 and 15.
18. Results. Page 8 para 1. I really like this random shuffle approach.
19. Results. Page 8. Para 3. I think the motif enrichment analysis needs more quantification. It's nice to see the motif plots in Fig 3H but would be good to see a table/bar chart of relative motif enrichment for each ChIP-seq group. I've had a look at Table S7 but there doesn't seem to be any distinction of different sample groups there.
20. Results. Figure 3. Panel F. It would be better to have actual p values rather than stars. EMM requires this as well as the test being stated in the legend.
21. Results. Figure 4. My reading of the text / figure is that this figure is entirely based on data from Massie et al EMBO J 2011 and also D'Antonio 2008. I think this needs to be clearer in the figure as panel A makes it look like novel data. Perhaps the editor will comment on whether this merits placement as a main figure given that this is the case. Perhaps better supplementary? Suggest that Panel A and D should state the method of stimulation (R1881) and deprivation (was this charcoal stripped media or bicalutamide treatment?). More recent papers have looked at prolonged growth of LNCaPs in charcoal stripped media.
22. Results. Figure 4. Panel C. It is not clear how these gene-sets are selected. Some Venn diagrams or something similar are needed to show this.
23. Results. Figure 4. Page 9 para 2. What are these three gene-sets? Cannot see a supp table with them.
24. Results. Figure 5. Panel A and D. It would be useful if the sample sets used here (and elsewhere) were actually named rather than just the GSE data number. It would improve recognition for the reader familiar with these datasets. I also think the scaling could be improved in these heatmaps. The colours are fairly blanched. Would suggest adjusting the colour distribution to more clearly demonstrate the enrichment transcript clusters (see colour scale in Fig 4B).
25. Results. Figure 5. Panel C and F. I think there are better ways to show these IPA outputs. Suggest taking the raw tabular outputs and turning into bar charts. Would then give more information in the same amount of space. "Above all, show the data"!
26. Results. Figure 6. I do not know anything about the "elastic double-loop cross validation" approach and trust that appropriate statistical expertise has been sought to review this aspect of the final element of this study.
27. Results. Figure 6 / Supp Fig 9. Why were these 6 cohorts chosen? Some comment on this is needed.
28. Results. Figure 6. Panel C. These principal component analyses nicely demonstrate the separation described. However there is not description or explanation of this approach is given in the text. Something needs to be introduced to page 10, penultimate paragraph.
29. Methods. I have not read the methods in great detail but they seem appropriate (apart from comments above re new versus public data).
30. Discussion. See general comments below.

Comments re typographical errors

1. Introduction. Final paragraph. FAIRE-seq is stated here for the first time here but as an abbreviation. Needed in full. Actually, I think this technique (probably not familiar to many, I hadn't heard of it before) should probably be explained in more detail in the text.
2. Results. Figure 2. The sequence of panels appearing in the text is really out of sync. This makes it quite a confusing figure and the text difficult to follow.
3. Results. Figure 4. Panel C. This is not labelled on the figure.
4. There are few typo errors.

General Comments

With the exception of the figure order issues mentioned above the manuscript is really well written. Congratulations. I think some of the statistical / geneset selection approaches need to be tightened or, at least, explained better in the text. In general, I think the claim that the authors make to have discovered a 'genomic pipeline for biomarker discovery' (discussion 1st para and abstract) is somewhat overstated and should be modified. Furthermore, I am not comfortable with the claim that this could be used to select men for adjuvant hormonal therapy. I think the most that can be claimed is that it highlights men that need to be monitored more closely after prostatectomy (with a view to subsequent treatment, whatever form that takes; RTx, Hormones, Taxane, Enza, AA, etc). If

anything, logic would suggest that an "acquired resistance classifier" could be used to highlight men for non-hormonal treatments. I look forward to seeing the authors' response.

1st Revision - authors' response 10 August 2015

Referee #1 (Remarks):

The manuscript by Zwart et al identified a distinct Androgen Receptor/chromatin binding profile between primary and resistant prostate cancers which in turn may be prognostic. Such a signature could be very important in placing patients into groups that require differential treatment.

Overall the data are provocative and the chromatin binding studies completed well. There are a number of important classifications and further experiments that are required in order that the reader can fully agree with the author's conclusions.

1. Are the authors driving forward a test that can be used a priori or only after surgery. IF the former, then the "clinical" pre-treatment staging must be used in addition to the signature in a multivariate analysis. If the latter, then additional pathologic and post-operative factors must be used in such analyses. Finally, if the authors are predicting ADT response, then a cohort that did or did not have early PSA failure after ADT was initiated should be used. At present the rationale for the choice of cohorts and the clinical utility is unclear.

We are aiming for a test that can be used after surgery to identify patients with high-risk of relapse. The newly included multivariate analyses were therefore based on post-operative factors (pathologic T stage, Gleason score, lymph node status), except PSA which is included as a pre-operative factor (Taylor et al., (GSE21034) and Boormans et al., (GSE41408)), see Supplementary Table S13. Since the acquired resistance geneset yields prognostic potential in both cohorts of primary prostate cancer patients (Figures 6A and C), these data indicate that the AR-resistance-driven processes may already be active in the primary tumor, driving prostate tumor progression and metastasis formation. To show that the acquired-resistance classifier can indeed separate androgen dependent from castration resistant tumors three cohorts (Tamura et al., (GSE6811) Sharma et al., (GSE28680), Terada et al.,(GSE21887)) were used (Figure 6D). With this, even though the classifier was originally designed as a prognostic biomarker, it may contain information on treatment response as well. To our knowledge, no gene expression datasets are available of cohort of patients that did or did not have PSA failure after ADT. The choice of the cohorts is now clarified in the text, and selection criteria are mentioned in the methods section.

2. The details of the clinical biostatistical studies and KM analyses are very unclear. What are the clinical factors used in the multivariate analyses for post-prostatectomy patients for each cohort and are these independent of known post-prostatectomy factors (margins, percent tumour, GS, PSA, T category, etc)?. For each cohort tested there should be a table of patient characteristics and the nature of the test and the MV statistical results.

A table with patient characteristics of the clinical cohorts used has been added to the manuscript (where available) (Supplementary Table S12). Furthermore, the nature of the statistical test used in KM analyses is now added to the main text and methods section. Multivariate analyses using standard prognostic factors, such as pathologic T stage, Gleason score, lymph node status and pre-operative PSA, have been added to the manuscript for each cohort (Supplementary Table S13).

Does the score aid prognosis across all NCCN/D'Amico pre-treatment prognostic groups? The authors should test for this association.

This is an excellent suggestion, and we would like to thank the reviewer for bringing this up. In the discovery cohort, we used the D'Amico prognostic classification to separate the patients into a low/intermediate and a high-risk group. Subsequently, we assessed the applicability of our 9-gene signature in these two patient subgroups, Figure 6B. In the low-intermediate group, we successfully separated an ultra-low risk group from an intermediate-risk group. In the high-risk D'Amico group, we could successfully separate the patients into an intermediate-risk and an ultra-high risk group based on our 9-gene signature. Statistical analyses showed that our 9-gene signature is an independent from D'Amico classification (Supplementary Table S14-S15).

The authors should complete a ROC-AUC curve for their signature to show utility above clinical variables alone.

An ROC-AUC analysis has now been performed and results are now included in the manuscript (Supplementary Table S14). The signature performed slightly better as compared to clinical variables (AUC 0.83 vs. 0.86, AUC of 0.902 when the signature is combined with the clinical parameters). Furthermore, the multivariate analysis of Supplementary Table S13 showed that our 9-gene signature is independent of the standard clinical variables.

Referee #2 (Comments on Novelty/Model System):

The authors have employed FAIRE-seq, ChIP-seq and transcript analysis to define patient tumour samples. ChIP-seq has been undertaken for the AR.

The authors comment on the significant variability in both AR peaks and FAIRE sites from sample-to-sample. There is little benchmarking of the AR data to other tissue datasets in the paper (site overlaps). There is no commentary on the mean size of the FAIRE sites in each sample or how size variation and variation in the number of sites may impact on motif enrichment analysis. More detailed discussion and cross-comparison is needed given the heavy emphasis on an integrative approach.

The average peak widths for both FAIRE and ChIP-seq have been added to the Supplementary Tables 2 and 5, respectively. The variable peak size does not affect motif search since a fixed window around the center of the peak is scanned by SeqPos, the tool used for motif discovery. The number of sites may indeed impact motif search: it affects the motif significance score. However, in the analysis we either do not compare motifs from different peak lists (in case of FAIRE-seq we focus only on the motifs that are found in primary tissue), or do not explicitly compare the scores that motifs get, but rather focus on the identity of the top identified motifs, providing qualitative analysis. The details of the motif analysis settings are now mentioned in the methods section.

Furthermore, we added more comparisons of FAIRE-seq data as well as our ChIP-seq data with other datasets from other reports (see below).

Referee #2 (Remarks):

The authors have generated maps of accessible chromatin, AR binding sites and transcript profiles from patient tissue samples. They argue that this represents an generalizable integrative approach for identifying cancer biomarkers. It is not entirely clear how the FAIRE data inform the other elements of the paper. Presumably in the absence of these data the authors, could based on other studies, have generated AR ChIP-seq data and generated a biomarker signature by integrating the sites with transcript profiles and comparison with public databases.

A number of points need to be addressed to make this less disjointed and more convincing as a single unified piece of work:-

1. The authors specify that the majority of the FAIRE-seq sites are in promoters. However the reads distribution in figure 2F is centered at the peaks centres and not at e.g. TSSs. This potentially introduces a bias in their analysis.

The message of Figure 2F is to emphasize the difference in the raw signal of chromatin accessibility in healthy prostate tissue as compared to different stages of prostate cancer, irrespective of the genomic locations. We have further focused on the transcriptional start site with our analysis in Figure 2H, where the peaks are separated into two sets; 1) promoters, defined as 3000 bp upstream of the transcriptional start site and 2) non –promoter regions. To illustrate that the vast majority of the FAIRE signal we observe at promoters is in fact enriched at TSSs (hence making us believe that we don't introduce a bias in our analyses) we now included a supplementary figure S1 that illustrates the enrichment of FAIRE-seq signal around the transcription start site of the genes affected.

2. From the legend of Fig 2H it is not clear which FAIRE-seq sites are taken into account? common?... only in one sample?

In Figure 2H, we used the FAIRE-seq sites that were present in at least two out of four primary tumors. This is now clarified in the figure legend.

3. The AR is a well known driver of PC. Is there anything from their FAIRE-seq data that would show a major involvement of other TFs on target genes with a prognostic significance? For example MYC and other TFs seem to occupy also a central area in their nodes-based analysis. Are the motifs consistent across different individuals within the same clinical group?

This is a very interesting question. To answer this, we have performed the overlap analysis between the FAIRE-seq peaks in the proximity of prognostic genes and a large collection of ChIP-seq datasets available through the ReMap tool (Griffon A et al. Nucleic Acids Res. (2015) 43(4):e27). For the 9 genes with prognostic significance, we found 8 accessible sites within 20 kb from the transcription start site, as assessed by FAIRE. For multiple transcription factors, including MYC, ERG, AR and FOXA1, binding sites were found at these FAIRE-sites. This information is now incorporated in Supplementary Table S10, and mentioned in the main text. Furthermore, as requested by the reviewer, we now also included motif analysis for each individual patient sample separately (Supplementary Table S4). These analyses illustrated that the motifs are largely consistent between the individuals.

4. Can the authors show overlaps of TFs binding with open regions? What is the overlap of AR binding sites with FAIRE sites as compared to others e.g. CTCF binding sites, ERG sites etc?

Analysis of AR, ERG and CTCF binding in FAIRE-seq peaks has already been included in the previous submission (See Figure 2H and Supplementary Figure S3B-D). As expected AR gives stronger signal at non-promoter FAIRE-seq as compared to promoter FAIRE-seq sites, given the fact that AR is an enhancer-enriched transcription factor. In contrast, CTCF is present at both enhancers and promoters that were accessible as assessed by FAIRE. The text has been updated to highlight this issue more clearly. We also added the analysis of FAIRE-seq open regions overlap with a collection of transcription factors with a ReMap tool (Griffon A et al. Nucleic Acids Res. (2015) 43(4):e27) and compared this overlap with the motifs scores (Supplementary Figure 2). We observed that CTCF was an outlier with a very high motif score and low overlap in binding, while both AR and ERG showed moderately high overlap and motif score. Interestingly, MYC showed a high overlap with the FAIRE-seq regions, which is of interest for future research on the role of this factor in transcription regulation of prostate cancer. This analysis is now added to the results section.

5. AR binding profiles differences were used to determine genes signature. Was the binding very heterogeneous compared to other papers?

As has been stated in the paper, number of AR binding sites varied between the patients and that is consistent with the previous data on ER binding in tumors (Ross-Innes et al. Nature. 2012; Jansen et al., Cancer Research. 2013), as well as AR binding in tumors: Sharma et al. found the number of binding sites varying from 300 to 8500 per tumor sample. This is now more explicitly mentioned in the main text.

Were there any site overlaps with other published studies? Could differential AR binding along their gene signature be applied also for the AR binding profile in CRPC ARBSs in Sharma et al?

The Sharma et al. signature was derived based on comparison of ChIP-seq data from tumors and cell lines and genes were further refined based on the changes in expression upon castration in xenograft models and expression change between untreated and CRPC tissue. Consequently, our signature has a completely different design, using ChIP-seq only from human tumor specimens.

Furthermore, the 'core' gene signature suggested by Sharma et al. was not trained for predicting survival and as such is not comparable to our signature. Nevertheless, we took advantage of the availability of the Sharma et al. clinical gene expression dataset and we demonstrate that a clear separation between untreated and CRPC samples can be obtained using the expression of the 9 genes in our signature. The data has been included in the original manuscript together with the xenograft data used in the same paper (Figure 6C). We now also included analysis of independent AR ChIP-seq data from clinical samples from Chen et al. (EMBO J. (2015) 12;34(4):502-16;primary PCa samples). We performed hierarchical clustering of these external data together with our tumor data based on the differential binding sites. 3 out of 4 primary tumors from Chen et al. clustered together with our primary tumor specimens, providing a benchmarking of our results with data from others. This is now included in Supplementary Figure S7 and stated in the text.

6. It would be useful to define the novel genes within the signature and to validate the the gene signature as a whole using qPCR in the clinical samples.

We have commented now on the novel genes (implying genes not described before in prostate cancer development or outcome) within the gene signature in the discussion section. qPCR validation of the 9 gene signature in the clinical samples was not possible at this stage, since for the tumors in which we performed AR ChIP-seq and FAIRE-seq, no tissue was left for expression analysis, and all remaining tissue is exclusively reserved for clinical purposes. Despite the fact that we cannot perform validation of the gene signature as a whole using qPCR, we do illustrate (Figure 6) our classifier as a robust means to stratify prostate cancer tumors on sensitivity to treatment and patients on outcome in five different gene expression datasets, namely:

- *Clinical expression data from 131 primary prostate tumors (Taylor et al., Cancer Cell 2010 Jul 13; 18)*
- *clinical expression data from 48 primary prostate tumors (Boormans et al., Int J Cancer 2013 Jul 15,133: 335)*
- *clinical expression data from 10 hormone-sensitive and 25 resistant tumors (Tamura et al., Cancer Res, 2007, 67; 5117)*
- *clinical expression data untreated and hormone-refractory tumors (Sharma et al., Cancer Cell, 2013, 23; 35)*
- *Mouse xenografts from Androgen-dependent, castration-resistant and castration-induced tumor regression (Terada et al., Cancer Res, 2010, 70; 1606)*

We also provided analysis of gene expression of individual genes at different stages of the disease in five extra independent clinical cohorts (Supplementary Figure S14).

With this, we believe that we have sufficiently illustrated the robustness of our classifier in a large number of patients, precluding the necessity of yet another mode of validation.

Finally in terms of the generalisability of the pipeline, it is clear that FAIRE-seq and ChIP-seq data can be compared to transcript profiles to find genes. However, for this approach to be generalizable the authors would need to show that the FAIRE approach is capable of identifying driving transcription factors in an unbiased manner, that ChIP-seq for those factors provides site maps that then enhance the filtering of differential transcripts to yield a more powerful biomarker signature. In this study at least as it is written it doesn't appear that FAIRE data alone has led the authors to the AR and it appears that the low and variable number of AR sites generated by ChIP-seq has restricted the number of candidate genes within the signature. The manuscript needs carefully restructuring to clearly illustrate the power and value of all of the presented elements in concert.

The main message of the story is that this approach can be used as a means and methods to identify transcription factors, its associated genes, and its corresponding gene signatures that enable the stratification of prostate cancer patients. Among all the transcription factors that were identified through the FAIRE-seq analysis, AR and many of its interacting transcription factors were

identified. Obviously, because of its clear role in prostate cancer, AR was a logical choice to pursue for further analyses. We do agree that we formally have not illustrated the generisability of the pipeline when it comes to picking any other transcription factor, and this statement is now clearly mentioned and included in the discussion section. Above all, we would like to position this work as a proof-of-principle, where generalizability still has to be proven in future follow-up work.

Regarding the limited number of genes identified, it may be good to mention that the number of genes we identified were in the same order of magnitude as described by Sharma et al, and it remains to be determined whether starting with a larger number of peaks would have provided us with a larger list differentially enriched peaks between primary and TURP samples.

Finally, we have altered the manuscript to illustrate the proof-of-principle concept better and to prevent overselling of the actual 'pipeline' concept, as was requested by another reviewer (#3).

Referee #3 (Remarks):

Summary

Stelloo and colleagues present a nice study of androgen receptor activity in four men with primary prostate cancer and three men with hormone relapsed disease, three with mets as well as some benign tissue. They use ChIP methods to identify binding sites in these different phases of the disease, including an approach called FAIRE-seq and attempt to demonstrate the distinct profiles. They construct a prognostic signature of nine genes and assess this in several public cohorts to demonstrate its clinical utility in predicting biochemical relapse.

Comments re Content

1. Abstract. It sounds in the abstract like all the data is taken from public datasets. Given that this is not the case (there are fresh ChIP studies of human material and cell lines) this should be made clear in the abstract. It fundamentally increases the power of the study and should right there to sell the story from the start!

We thank the reviewer for pointing this out. We have updated the abstract accordingly, to further highlight the fact that we have in fact performed a large series of original experiments in this work.

2. Introduction. First para, second sentence. Patients are normally treated with "prostatectomy and/or radiotherapy" rather than the other way round.

We have corrected this.

3. Introduction. Fourth para, first sentence. This is not always the case. Men with recurrence are normally offered salvage radiotherapy after prostatectomy or locally advanced disease are often offered radiotherapy plus hormones or indeed prostatectomy and adjuvant RTx.

The sentence has been changed to: Patients with failed salvage therapy or metastatic prostate cancer are treated with ADT as a first-line palliative treatment.

4. Introduction. Fourth para, 3rd sentence. Needs a reference. As do subsequent statements.

References are now included to support these statements.

5. Introduction. Fourth para, final sentence. There are actually several papers that present "biomarkers" (signatures actually) that enable identification of prostate cancer patients at high risk of relapse (e.g. Ramos-Montoya et al EMM 2014 HES66/E2F1/AR signature, Irshad et al Nat Comms 2014 "indolence" signature, Lalonde et al Lancet Onc 2014 copy number signature).

We fully agree with the reviewer. We have updated the text accordingly, now stating that multiple different biomarkers/signatures have been developed to identify high-risk patients, and multiple examples thereof are being referenced. Nonetheless, it should be mentioned that no such signature-based biomarkers are currently adopted in routine clinical practice, which is now also more clearly stated in the text.

© EMBO

6. Introduction. 5th para. 4th sentence. This comment about resistance to inhibitors needs a reference. (e.g. Antonarkis et al, NEJM).

The reference is now included.

7. Introduction. 5th para. Final sentence. There have been some nice reviews of these mechanisms of resistance which should probably be referenced (instead of individual studies). See Pienta et al Ca Cancer J Clin 2005 or most recently Lamb et al BJUI 2014.

These suggested review by Lamb et al is now included in the references.

8. Results. Figure 1. Ok

great!

9. Results. Figure 2. Am confused by numbers here. Are there three or four primary? There are three in text. Four on figure 2C. Four on table S1.

We apologize for the mistake and thank the reviewer for pointing this out. There are four primary tumors, and this is now corrected in the main text.

10. Results. Figure 2. 2nd para page 6. This figure needs more numbers I think. What are the stats for the quantitative statements made here? A statement is made about there being an increase in chromatin accessibility in tumour samples but no numbers or tests are presented. Is this really true? There are only four samples.

We have added statistics into the text and substituted panel C with the boxplot that illustrates the distribution of normalized read counts in peaks (reads per kilobase per million, RPKM; Figure 2D) in different clinical groups. As can be appreciated from the figure and supporting statistical analyses, a significant increase in chromatin accessibility is indeed observed.

11. Results. Figure 2. Panel B. These averaged wig file plots should all be on the same y axis. At the moment it is hard to compare the peak calls visually. In fact, it looks like there is no difference at all at the moment whereas, if the axis is changed, the difference will be more apparent (and hopefully send a more powerful message!). Also, why are these four representative peaks chosen?

These snapshots were chosen to illustrate the presence of the FAIRE-seq signal in all the studied sample groups. We found no clear difference in their signal among different stages of the disease and the peaks are not meant for direct comparison, but merely to illustrate how the raw data looks at 4 randomly picked regions. This is now more clearly mentioned in the text. Nevertheless, in order to avoid confusion, we changed the figure and have now normalized all the y-axes to millions of sequenced reads and set the y-axes limits to the same value for all the samples.

12. Results. Figure 2. Panel C. needs stats.

We have added statistics into the text and substituted panel C with the boxplot that illustrates the distribution of normalized read counts in peaks (reads per kilobase per million, RPKM) in different

© EMBO

clinical groups (Figure 2D).

13. Results. Figure 2. Panel E. There seems to be much more promoter binding in benign compared to tumour, which instead has much more intron and distal intergenic binding. I don't think this is commented on at all in the text.

This conclusion is correct, and we indeed omitted this statement in the original submission. Now, it is clearly stated in the text that tumor samples show more chromatin accessibility at both intron and distal intergenic regions as compared to benign tissue, where more promoter accessibility is found.

14. Results. Figure 2. Panel G. What was the input list for IPA. Was it table S8? Either way, this needs to be made clear in the text.

Supplementary table S3 (list of genes corresponding to motifs identified in FAIRE-seq data from primary PC tumors) was used as the input for IPA. This has been now added to the text, and we apologize for not clearly mentioning this before in the original submission.

15. Results. Figure 3. Panel B. Same as above.

We changed the panel with ChIP-seq snapshots as described above for Figure 2B, namely, we normalized the read count to the million of sequenced reads and set the y-axis limits to the same values for all the samples.

16. Results. Figure 3. Panel C. I think this could be displayed more clearly with a subcluster plot. See Ramos Montoya et al, EMM 2014 Figure 2D for an example.

We believe that our initial figure is very similar in essence to the mentioned Figure 2D from Ramos-Montoya as it is a similar heatmap. To enhance clarity and to ensure better interpretation of the figure, we now clarified the labels and changed the colors of the heatmap to correspond to the clustering according to the clinical sample identity.

17. Results. Figure 3. Panel E. The key needs to be improved on here so that all the colours are specified. There seem to be more than two colours on each plot. Or if these are replicates then perhaps they should be averaged. See Ramos-Montoya et al, EMM 2014 Figure 2B for an example.

We double-checked and validated that only two colors are present on each plot: red and green. For clarity, we have now increased the line width to enhance the quality of the figure. Each line in panel 3E represents a single ChIP-seq sample (two of them come from the same patients, while the others are independent patients). We believe it is important to show every sample and not average profiles within the group in order to show biological variation within the clinical groups.

Figure 2A and C also gives an example of points mentioned above in 11 and 15.

Figure 2A shows a schematic representation of the experiment and does not contain any data. Panel 2C has now been substituted by a more quantitative analysis in Figure 2D, as mentioned in 12.

18. Results. Page 8 para 1. I really like this random shuffle approach.

© EMBO

We appreciate the reviewer's comment, and are delighted to hear the approach is well received.

19. Results. Page 8. Para 3. I think the motif enrichment analysis needs more quantification. It's nice to see the motif plots in Fig 3H but would be good to see a table/bar chart of relative motif enrichment for each ChIP-seq group. I've had a look at Table S7 but there doesn't seem to be any distinction of different sample groups there.

We fully agree. To resolve this issue, we added a heatmap that illustrates the enrichment of motifs in different sample groups (Supplementary Figure 9) as well as a new table with all the motifs scores from different sample groups (Supplementary Table S7).

20. Results. Figure 3. Panel F. It would be better to have actual p values rather than stars. EMM requires this as well as the test being stated in the legend.

We have now added the p-values to the figure and provided the details of the test used in the figure legend.

21. Results. Figure 4. My reading of the text / figure is that this figure is entirely based on data from Massie et al EMBO J 2011 and also D'Antonio 2008. I think this needs to be clearer in the figure as panel A makes it look like novel data. Perhaps the editor will comment on whether this merits placement as a main figure given that this is the case.

Perhaps better supplementary? Suggest that Panel A and D should state the method of stimulation (R1881) and deprivation (was this charcoal stripped media or bicalutamide treatment?). More recent papers have looked at prolonged growth of LNCaPs in charcoal stripped media.

Obviously, we have not written this section clear enough. The transcriptomic data were stored by Massie et al. and D'Antonio et al on the NCBI GEO repository. What we have done, is that we specifically selected the concise list of 158 genes from their entire set of over 47,000 transcripts that we identified to be proximal to a differential enriched AR binding site between primary and TURP samples. Such a focused re-analyses and funneling of publically available data is (in our opinion) surely novel enough to warrant inclusion in the main figures. This figure is above all original and based on our thoroughly selected genelist. The mode of stimulation and deprivation (charcoal stripped media) is now included in the figure itself and the legends. As the reviewer rightfully states, more recent papers have looked at prolonged growth of LNCaP cells in charcoal-stripped media, but for the purpose as applied here, the current dataset sufficed in our opinion.

22. Results. Figure 4. Panel C. It is not clear how these gene-sets are selected. Some Venn diagrams or something similar are needed to show this.

The genes were grouped based on their temporal profiles as identified using temporal clustering of gene expression using Short Time-series Expression Miner (STEM, J. Ernst, Z. Bar-Joseph. BMC Bioinformatics, 7:191, 2006), as has been described in the methods. We added the description to the results section and to the figure legend, in order to more clearly explain how the genes were selected. Importantly the temporal signal of expression is a key aspect is gene-set definition, which cannot be visualized as such in a Venn diagram.

23. Results. Figure 4. Page 9 para 2. What are these three gene-sets?

Cannot see a supp table with them.

The gene sets have now been added in the Supplementary Table S9.

24. Results. Figure 5. Panel A and D. It would be useful if the sample sets used here (and elsewhere) were actually named rather than just the GSE data number. It would improve recognition for the reader familiar with these datasets. I also think the scaling could be improved in these heatmaps. The colours are fairly blanched. Would suggest adjusting the colour distribution to more clearly demonstrate the enrichment transcript clusters (see colour scale in Fig 4B).

As suggested by the reviewer, we now specified the name of the dataset (first author,et al, year) for all the Figures where publically available datasets have been used. Furthermore, we improved the color scheme in Figure 5.

25. Results. Figure 5. Panel C and F. I think there are better ways to show these IPA outputs. Suggest taking the raw tabular outputs and turning into bar charts. Would then give more information in the same amount of space. "Above all, show the data"!

This is a great suggestion: we now generated barplots that represent the results of the IPA analysis and included these in Figure 5E.

26. Results. Figure 6. I do not know anything about the "elastic double-loop cross validation" approach and trust that appropriate statistical expertise has been sought to review this aspect of the final element of this study.

27. Results. Figure 6 / Supp Fig 9. Why were these 6 cohorts chosen? Some comment on this is needed.

The cohorts were selected based on the following criteria:

-frozen tissue

-complete datasets, where the (most of) the 9 genes were present

-Large enough patient series

- either the follow-up data present, or response/disease stages defines

These details are now included in the methods section.

We have included this description now in the methods section, fully explaining the selection criteria for these 6 cohorts.

28. Results. Figure 6. Panel C. These principal component analyses nicely demonstrate the separation described. However there is not description or explanation of this approach is given in the text. Something needs to be introduced to page 10, penultimate paragraph.

As requested by the reviewer, a description of the analysis is now included in this paragraph, explaining the methodology better.

29. Methods. I have not read the methods in great detail but they seem appropriate (apart from comments above re new versus public data).

Figures are changed to indicate publically datasets (First author, GEO number), in order to make it more clear which data is new and which is public.

30. Discussion. See general comments below.

Comments re typographical errors

1. Introduction. Final paragraph. FAIRE-seq is stated here for the first time here but as an abbreviation. Needed in full. Actually, I think this technique (probably not familiar to many, I hadn't heard of it before) should probably be explained in more detail in the text. 2. Results. Figure 2. The sequence of panels appearing in the text is really out of sync. This makes it quite a confusing figure and the text difficult to follow.

FAIRE-seq is written out in full in the introduction and explained more in detail along with the accompanying Figure 2A. Furthermore, the sequence of the panels of Figure 2 has now been updated so that it follows the order as mentioned in the main text.

3. Results. Figure 4. Panel C. This is not labelled on the figure.

Panel C is now correctly labeled in the figure.

4. There are few typo errors.

We have gone through the text extensively and tried to remove all typo's.

General Comments

With the exception of the figure order issues mentioned above the manuscript is really well written. Congratulations. I think some of the statistical / geneset selection approaches need to be tightened or, at least, explained better in the text. In general, I think the claim that the authors make to have discovered a 'genomic pipeline for biomarker discovery' (discussion 1st para and abstract) is somewhat overstated and should be modified. Furthermore, I am not comfortable with the claim that this could be used to select men for adjuvant hormonal therapy. I think the most that can be claimed is that it highlights men that need to be monitored more closely after prostatectomy (with a view to subsequent treatment, whatever form that takes; RTx, Hormones, Taxane, Enza, AA, etc). If anything, logic would suggest that an "acquired resistance classifier" could be used to highlight men for non-hormonal treatments. I look forward to seeing the authors' response.

We thank the reviewer for the supportive feedback and helpful comments. We agree with the reviewer that the statement of 'discovery of a genomics pipeline' is indeed somewhat overstated, since we simply combined technologies instead of designing new technologies ourselves. This is now more clearly mentioned in the text, and the 'development of a genomics pipeline' is trimmed down.

Furthermore, we fully agree that our classifier is above all aimed to identify patients with a high risk of relapse, and this could be used to monitor patients more closely after prostatectomy. This is now clearly included in the main text and discussion section and we thank the reviewer for pointing this out. Future studies will be aimed to determine whether our classifier could potentially be applied to identify patients selectively responding to a given therapy, being either hormonal or non-hormonal. Since our PCA analysis did successfully stratify hormone refractory from primary

samples (Figure 6D), a certain level of hormone-response prediction seems to be represented in the classification, but more research on this matter should be performed to fully unravel the matter of treatment response prediction versus merely being prognostic. Also this is now incorporated in the discussion section.

2nd Editorial Decision 28 August 2015

Thank you for the submission of your revised manuscript to EMBO Molecular Medicine. We have now received the enclosed reports from the referees that were asked to re-assess it. As you will see the reviewers are now globally supportive, although Reviewer 3 lists a few minor remaining items for your action. I agree with the Reviewer that Figure 3 can be de-cluttered by moving the p-values to the legend.

In the likely event of acceptance, you will be asked to fulfill a number of editorial requirements.

I would like to suggest alternative titles to increase impact and readability: "Androgen Receptor profiling establishes prostate cancer outcome"or
"Prostate cancer outcome from patient androgen receptor profiling". Please feel free to suggest further along these lines.

I look forward to reading a new revised version of your manuscript as soon as possible.

***** Reviewer's comments *****

Referee #1 (Remarks):

All issues now addressed
Thank you

Referee #2 (Comments on Novelty/Model System):

The authors have presented satisfactory responses to rebuttal points. It remains to be seen how technically replicable the approach is or widely adopted the signature will be.

Referee #2 (Remarks):

The authors have addressed the points raised.

Referee #3 (Remarks):

Thank you for your thorough and detailed response to all the comment made. I accept the rebutted comments (e.g. points 16, 17, 21,22).

Please see below a few further items for clarification (which I am happy for the editor to manage).

1. I cannot see any addition to the abstract that makes clear that these are novel clinical samples being analysed in a wet-lab setting. I am still not sure this is clear as the only clear mention of data-source is the "publicly available transcriptomic and clinical datastreams" in the third last line of the abstract.

2. Figure 2. I think there is some wasted space bottom right of the figure which is slightly more pronounced since the changes you've made. Can I suggest that you re-jig the order/size of panels here in the figure to make sure that space is used (the peak heatmaps could be widened of the pie charts made bigger (could all be done while keeping the letter order the same)

3. I suggest with the bar chart in Figure 3 that you put a key with letters a-h etc and the p values themselves in the legend. It's good that EMM require this as it makes the data more transparent, but can make the figure clunky!

As a general comment, it was quite hard to see what had been changed in the manuscript. Perhaps useful to leave some tracked changes in an uncleaned version for our benefit?

Good work!

2nd Revision - authors' response 01 September 2015

Referee #1 (Remarks):

All issues now addressed

Thank you

Referee #2 (Comments on Novelty/Model System):

The authors have presented satisfactory responses to rebuttal points. It remains to be seen how technically replicable the approach is or widely adopted the signature will be.

Referee #2 (Remarks):

The authors have addressed the points raised.

Referee #3 (Remarks):

Thank you for your thorough and detailed response to all the comment made. I accept the rebutted comments (e.g. points 16, 17, 21,22).

Please see below a few further items for clarification (which I am happy for the editor to manage).

1. I cannot see any addition to the abstract that makes clear that these are novel clinical samples being analysed in a wet-lab setting. I am still not sure this is clear as the only clear mention of data-source is the "publicly available transcriptomic and clinical datastreams" in the third last line of the abstract.

The abstract has now been updated, more explicitly highlighting the fact that we have generated original and novel genomics data in prostate tissue specimens. In the third (we assessed..) and final

last sentence (generation of.. integration with..) of the abstract we now clearly emphasize the original and publically available data.

2. Figure 2. I think there is some wasted space bottom right of the figure which is slightly more pronounced since the changes you've made. Can I suggest that you re-jig the order/size of panels here in the figure to make sure that space is used (the peak heatmaps could be widened of the pie charts made bigger (could all be done while keeping the letter order the same)

The Figure layout has been updated, now making more efficient use of the space available.

3. I suggest with the bar chart in Figure 3 that you put a key with letters a-h etc and the p values themselves in the legend. It's good that EMM require this as it makes the data more transparent, but can make the figure clunky!

The reviewer suggestion has now been implemented, moving the individual p values to the legends, putting a key in the actual figure.

As a general comment, it was quite hard to see what had been changed in the manuscript. Perhaps useful to leave some tracked changes in an uncleaned version for our benefit?

Good work!

We fully agree that 'track change' makes it considerably easier to assess changes in the manuscript. To our knowledge, track changes is not supported by the PDF conversion tool provided by EMBO mol med. If considered of added value, we would be delighted to email a separate Word file in 'track change' to the editorial office for your consideration.
